# Health and Socio-Economic Impacts of Climate-Related Displacement in Bangladesh’s Chars: Causal Evidence From a Household Survey

**DOI:** 10.3389/ijph.2026.1608475

**Published:** 2026-03-11

**Authors:** Juan A. de Castro, Laurentiu Guinea

**Affiliations:** 1 Department of Economics, Universidad Nebrija, Madrid, Spain; 2 Universidad Complutense de Madrid, Instituto Complutense de Análisis Económico (ICAE), Madrid, Spain

**Keywords:** causal inference, disease burden, displacement, economic vulnerability, household resilience

## Abstract

**Objectives:**

To assess health and socio-economic impacts of climate-related displacement in North-East Bangladesh chars and examine links between non-governmental services, disease burden and migration.

**Methods:**

We analysed a household survey of 480 women aged 15–55 from nine intervention and three comparison chars, collected between March and June 2022. Using a quasi-experimental framework and estimators of the average treatment effect, we compared displaced and non-displaced households and households in chars with and without Friendship health and education services. We constructed indices of disease burden, migration and socio-economic conditions, each scaled 0–100.

**Results:**

Displaced households had lower disease burden scores than non-displaced households after adjusting for socio-economic covariates. This pattern is consistent with improved access to services among some displaced groups, but may also reflect reporting differences and selection into the observed displaced population. Migration intensity was higher in chars where Friendship operates than in comparison chars, suggesting programme placement in areas with stronger migration pressures.

**Conclusion:**

Climate-related displacement interacts with service access, vulnerability and selection in complex ways; targeted interventions can reduce disease burden but do not necessarily lower migration pressures.

## Introduction

Recent years have seen a surge in displacement affecting the poorest. In low-income nations like Bangladesh, frequent disasters–flooding and river erosion–force communities to leave their homes, worsening health risks and socio-economic inequalities (e.g., [1–3]). Projections warn that by 2050 one in seven Bangladeshis could be displaced [4], spurring public health crises with outbreaks of diarrhea, skin diseases, respiratory ailments, malaria, and dengue [5].

Chars–Bangladesh’s riverine islands–face acute risks as displacement disrupts livelihoods and worsens water and sanitation [6]. Recurring floods and erosion interrupt stable housing, healthcare, and economic opportunities [7, 8], and unlike permanent migration, temporary displacement keeps families within national borders amid ongoing disruptions [9, 10]. Addressing these issues demands targeted health and education interventions and coordinated efforts by governments, NGOs, and communities. NGOs like Friendship NGO bridge resource gaps by providing disaster preparedness, health services, and aid that bolster disease prevention, hygiene, and access to essential care (e.g., [11, 12]).

This study explores the health and socio-economic impacts of temporary displacement on households while assessing Friendship NGO’s interventions, thus contributing to research on displacement effects and NGO roles. Unlike prior work that focuses on permanent or cross-border migration [10, 13, 14], our focus is on temporary recurrent displacement–where families remain nationally but face persistent instability and environmental shocks. Analysis of survey data from treatment and control chars in North-East Bangladesh reveals a concerning prevalence of communicable diseases, including diarrhea, dysentery, and malaria, among displaced households.

Our study employs methods tailored to our quasi-experimental design [15]. Using causal inference frameworks [16–19] and guidance from [20, 21], we estimate the Average Treatment Effect (ATE) to measure the impact of temporary displacement–caused by flooding and erosion–by comparing displaced and non-displaced groups while controlling for biases. For robustness, we apply Regression Adjustment [22], Inverse Probability Weighting [23], Augmented IPW [24], IPWRA [25], and Nearest Neighbor Matching [26].

We follow [27] to build five indices of household wellbeing: the Migration Household Index (MHI) for migration dynamics; the Disease Burden Index (DBI) for health severity (with higher scores indicating poorer health); the Household Resource Index (HRI) for wealth; the Educational Attainment Index (EAI) for education levels; and the Hygiene Household Index (HHI) for sanitary practices and access to essentials. These indices are used in two models.

Model 1 examines the effect of displacement on DBI by comparing displaced and non-displaced households. Although we initially hypothesized that displaced households would exhibit higher DBI scores, our results reveal significantly lower scores among the displaced. This counterintuitive outcome is consistent with a combination of mechanisms. On the one hand, displaced households, especially in treatment chars, may benefit from greater exposure to Friendship NGO health services and other providers, which can improve prevention, diagnosis and treatment. On the other hand, displacement may alter care-seeking behaviour and how symptoms are perceived and reported, generating systematic reporting differences between displaced and non-displaced groups. In addition, selection mechanisms–including a possible form of “survivor bias”, whereby more resilient households are more likely to remain observable among the displaced–could lead us to underestimate the true health risks faced by the most vulnerable, who might experience more severe outcomes or exit the sample altogether.

Model 2 evaluates how residence in chars where Friendship NGO operates is associated with the Migration Household Index (MHI), which captures household migration dynamics. As detailed below, our estimates show that treatment chars have systematically different migration patterns–with higher MHI values on average–than control chars. This pattern is consistent with Friendship prioritising chars that are already experiencing stronger migration pressures and highlights the need for caution when interpreting cross-sectional associations between NGO presence and migration as causal effects.

The rest of the paper is structured as follows: Section *Methods* describes the survey design, the methodology for constructing the indexes, the treatment effect estimation methodologies and introduces the theoretical models. Section *Results* presents the estimation results, interprets the findings. Finally, Section *Discussion* discusses the study’s limitations and concludes.

## Methods

Data were collected by Friendship NGO via a household survey (March–June 2022) in North-east Bangladesh chars. The survey included nine treatment chars with active health and education interventions (Bajra Diyar Khata, Batikamari, Char Jatrapur, Kawa Bada, Khamar Bashpata, Khamar Holokhana, Kheyar Alga, Korai Barishal, South Sannasir Char) and three control chars (Chor Garuhara, Datiar Char, Moheshbandi).

A total of 480 women (aged 15–55) participated, offering insights into health, migration, wealth, and education (see Supplementary Table A.1). Detailed survey design that includes respondent selection, questionnaire design, implementation, and ethics is provided in the Supplementary Appendix (see Supplementary Appendix B for migration drivers and Supplementary Appendix C for methodological details).

Our survey examines migration drivers in Northeast Bangladesh’s chars (see Supplementary Table B.1). River erosion is the primary factor–displacing households, degrading arable land, and disrupting livelihoods–while economic decline forces families toward urban areas for better opportunities.

Poverty and landlessness, compounded by recurrent shocks, further drive migration, with socio-cultural issues playing a lesser role. These results highlight the intertwined environmental, economic, and social forces shaping migration and the need for integrated adaptation and development strategies.

We constructed five indices from survey responses to quantify household conditions: the Migration Household Index (MHI) for displacement, the Disease Burden Index (DBI) for illness severity, the Wealth Household Index (WHI) for assets, the Educational Attainment Index (EAI) for schooling, and the Hygiene Household Index (HHI) for sanitation and water access. These indices structure our analysis of socio-economic vulnerabilities and displacement impacts for further empirical study.

### Migration Household Index (MHI)

The Migration Household Index (MHI) measures household mobility by capturing migration frequency, duration, and type, along with its drivers. It uses survey data on whether households have migrated, how often and how long, and the reasons–whether environmental (flooding, erosion), economic, or social.

The Migration Household Index is defined in Equation 1 and normalized in Equation 2.

$$\text{MHI}=\frac{\sum_{i=1}^{N}\left({\text{Weight}}_{\text{t},i}\times {\text{Frequency}}_{i}\times {\text{Prop}}_{\text{m},i}\right)}{\text{Total Household Members}},$$ (1)

where: N represents the total number of migration events reported by the household, ${\text{Weight}}_{\text{t},i}$ represents the weight assigned to each type of migration based on its socio-economic impact (e.g., permanent migration may carry a higher weight than seasonal migration), ${\text{Frequency}}_{i}$ represents the number of times a specific type of migration occurred ${\text{Prop}}_{\text{m},i}$ : The proportion of household members involved in each migration event. Total Household Members : Total number of individuals in the household.

The raw MHI is then normalized to ensure comparability across households:

$$\text{Normalized MHI}=\frac{\text{Raw MHI}-\text{Minimum MHI}}{\text{Maximum MHI}-\text{Minimum MHI}}\times 100,$$ (2)

where *Minimum MHI* and *Maximum MHI* represent the observed minimum and maximum values of the raw index in the dataset.

#### Interpretation of MHI

The MHI standardizes household migration. Higher values indicate frequent or impactful moves due to environmental or socio-economic pressures, while lower values suggest stability. It is key for understanding migration in Northeast Bangladesh chars, where flooding and erosion force relocations.

Supplementary Appendix C.1 details the index with histograms (Supplementary Figure C.1) comparing treatment and control chars and scatter plots (Supplementary Figure C.2) on demographic trends.

### Disease Burden Index (DBI)

The Disease Burden Index (DBI) measures the prevalence and severity of health conditions in households from Northeast Bangladesh’s chars, standardizing assessment by considering both the share of affected members and illness intensity.

Following [27], we define the Disease Burden Index (DBI) for household i . The Disease Burden Index is computed using Equations 3, 4 and normalized in Equation 5.

$$DBI_{i}=\frac{T_{i}}{N_{i}}\times 100,$$ (3)

where $T_{i}$ is the total illness types reported and $N_{i}$ is the household size. The index is positive if any member is ill, capturing both the proportion of ill members and their illness severity. Defining $n_{i}$ as the number of reported illness types, we decompose:

$$DBI_{i}=\frac{n_{i}}{N_{i}}\times \frac{T_{i}}{n_{i}}\times 100=dbi_{i}^{p}\times dbi_{i}^{s}$$ (4)

With $dbi_{i}^{p}$ measuring the ill proportion and $dbi_{i}^{s}$ indicating severity (see Supplementary Appendix C.2).

#### Weights and ICD-10 Integration in the DBI

We incorporate multiple dimensions into the Disease Burden Index (DBI) to capture the prevalence, severity, duration, and impact on daily functioning and quality of life of various conditions. We begin by identifying relevant diseases using survey questions such as “Did your family members suffer any diseases within the last 6 months?” and “Are you or your family members affected by any infectious/communicable diseases?” This process captures a range of infectious and non-infectious conditions–including malaria, diarrhea, typhoid, and COVID-19 – which are then mapped to their corresponding ICD-10 codes for global standardization.

Weights are assigned based on each disease’s prevalence, severity (in terms of disability or impairment), and quality-of-life impact (measured by disruptions to daily activities). More prevalent or severe diseases (e.g., pneumonia or COVID-19) receive greater weights than milder conditions like muscle soreness or skin diseases. Finally, the DBI for each individual or household is calculated by aggregating these weighted scores into a single measure of overall disease burden.

A higher DBI indicates deteriorating health conditions, as it corresponds to a greater burden of disease within the household. To facilitate comparability, the DBI is normalized to range between 0 and 100 using the following formula:

$$\text{Normalized DBI}=\frac{\text{Raw DBI Value}}{\text{Maximum Possible DBI}}\times 100.$$ (5)

Table 1 lists the diseases included in the DBI along with their corresponding ICD-10 codes, facilitating integration with broader health datasets and ensuring adherence to international classification standards.

**TABLE 1:** Weights and ICD-10 codes for all diseases in the study: Bangladesh, 2022 (Friendship NGO household survey, Bangladesh, 2022).

Disease	Severity/Impact level	Weight	ICD-10 code
Malaria	Very high (fatal if untreated)	4.5	B50–B54
ARI/Pneumonia	Very high (life-threatening)	5.5	J10–J18
COVID-19	Very high (pandemic, systemic impact)	5.0	U07.1
Blood dysentery	High (serious, requires care)	4.5	A03
Typhoid	High (systemic, severe impact)	4.5	A01
Jaundice	High (can indicate liver damage)	3.5	R17
Hepatitis (A, C, D)	High (liver-related, systemic impact)	4.0	B15, B16.0, B17.1
Cholera	High (acute, potentially fatal)	4.5	A00
Fever and cough	Moderate (common, mild)	1.5	J00–J06
Diarrhea	Moderate (common, treatable)	3.5	A09
Muscle soreness	Low (minor health impact)	2.0	M79.1
Worms	Low (minor health impact)	2.0	B65–B83
Skin diseases	Low (non-life-threatening)	2.0	L00–L99
Others	Varies based on specific disease	0.5	Various

To make the weighting scheme more transparent, we followed a three-step procedure. First, each condition listed in Table 1 was assigned to one of four clinical impact tiers—very high, high, moderate or low—based on its potential fatality risk, likelihood of serious complications, and typical intensity of care required. This classification draws on the burden disease literature for similar settings and on consultations with medical staff from Friendship NGO regarding the conditions most commonly observed in the chars. Second, we mapped these qualitative tiers into a monotone numerical scale between 0.5 and 5.5, so that very high impact conditions such as ARI/pneumonia or COVID-19 receive the largest weights, high impact conditions such as cholera, typhoid or malaria receive slightly smaller but still elevated weights, and moderate and low impact conditions (for example, fever and cough, worms or minor skin diseases) receive lower weights. The exact values are chosen to preserve the ordering across tiers while avoiding extremely large differences that would make the index overly sensitive to a single reported diagnosis. Third, we verified that the assigned weights are broadly consistent with reported days of illness and with patterns of care-seeking and hospitalization reported in the survey.

Because any index of this type inevitably involves judgment, we conducted a set of sensitivity analyses to assess whether our main results depend on the particular choice of weights. In Supplementary Appendix C.2 we re-construct the DBI under several alternative schemes: (i) equal weights for all disease categories; (ii) a coarser four-level severity scale that compresses the range of differences between conditions; and (iii) a specification that excludes low-impact conditions such as minor skin diseases and muscle soreness. We then re-estimate Model 1 using each of these alternative DBI measures. As summarised in Supplementary Table C.3, the estimated effect of displacement on disease burden remains negative and statistically significant across these specifications, and the magnitudes are of a similar order to our baseline estimates. This suggests that our substantive conclusions are not driven by a particular parametrisation of the DBI weights.

Supplementary Appendix C.2 presents DBI histograms by treatment (Supplementary Figure C.3) and displacement status (Supplementary Figure C.4), enabling clear comparisons of health vulnerabilities between households in treatment chars (where Friendship NGO operates) and control chars, as well as between displaced and non-displaced groups.

Scatter plots of DBI by treatment (Supplementary Figure C.5) and displacement (Supplementary Figure C.6) further reveal how age affects health outcomes, highlighting differences among younger and older members.

### Wealth Household Index (WHI)

The Wealth Household Index (WHI) combines three wealth dimensions: land ownership (value of farmland, homestead, and rented/mortgaged land; see Supplementary Table C.3), household goods (furniture, appliances, transportation), and livestock (e.g., cows, goats, poultry). Values are based on market prices summarized in Supplementary Table C.3 (in Bangladeshi Taka).

We illustrate wealth distribution using WHI histograms by treatment (Supplementary Figure C.7) and displacement status (Supplementary Figure C.8), comparing treatment chars (where Friendship NGO operates) to control chars and displaced to non-displaced households. Scatter plots (Supplementary Figures C.9, C.10) further explore the relationship between age and wealth.

### Hygiene Household Index (HHI)

The Hygiene Household Index (HHI) measures household hygiene by combining data on sanitation behaviors, infrastructure, cultural practices, water usage, and toilet facilities. It uses binary variables (e.g., handwashing, waste disposal) alongside categorical assessments of water source, accessibility, and toilet quality (see Supplementary Table C.4).

Histograms of the HHI–disaggregated by treatment (Supplementary Figure C.11) and displacement (Supplementary Figure C.12)– compare hygiene conditions between treatment chars (Friendship NGO beneficiaries) and controls, and between displaced and non-displaced households. Scatter plots (Supplementary Figures C.13, C.14) further examine how age affects hygiene practices.

### Educational Attainment Index (EAI)

The Educational Attainment Index (EAI) measures household education by weighting each member’s highest education level adjusted for age (see Supplementary Table C.5), allowing comparisons across age groups. Histograms by treatment (Supplementary Figure C.15) and displacement (Supplementary Figure C.16) compare households in treatment chars (Friendship NGO) versus controls and displaced versus non-displaced households, while scatter plots (Supplementary Figures C.17, C.18) highlight generational differences in educational access.

### Summary Statistics for the Indexes

The survey results are summarized through the construction of five key indices–Disease Burden Index (DBI), Wealth Household Index (WHI), Migration Household Index (MHI), Hygiene Household Index (HHI), and Educational Attainment Index (EAI) – to evaluate health and socio-economic conditions. Table 2 provides a compact overview of each index, including its main dimension, scale and the interpretation of higher values. Table 3 then reports descriptive statistics for these indices in the full sample, while Supplementary Tables C.7, C.8 present separate descriptive statistics by treatment status and displacement, providing a comparative overview of the treatment and control chars, as well as displaced and non-displaced households.

**TABLE 2:** Overview of constructed household indices: Bangladesh, 2022 (Friendship NGO household survey, Bangladesh, 2022).

Index	Main dimension	Direction^a^	Interpretation of higher values
DBI	Health	Worse	Greater overall disease burden in the household (higher prevalence and severity of reported conditions)
MHI	Migration	Worse	More intense or frequent migration and displacement events, indicating higher mobility and instability
WHI	Economic resources	Better	Higher household wealth, combining land, durable goods and livestock holdings
HHI	Water, sanitation and hygiene	Better	Better hygiene practices and WASH infrastructure (e.g., safer water sources, improved sanitation)
EAI	Education	Better	Higher overall educational attainment of household members, adjusted for age

All indices are normalised to range from 0 to 100.

^a^ ”Worse” indicates that higher values correspond to worse outcomes (higher disease burden or more intense migration); “Better” indicates that higher values correspond to better outcomes (more wealth, improved hygiene, or greater educational attainment).

**TABLE 3:** Descriptive statistics for constructed indexes: Bangladesh, 2022 (Friendship NGO household survey, Bangladesh, 2022).

Index	Mean	p10	p25	p50	p75	p90	SD	Min	Max	N
WHI	5.85	0.00	0.40	2.42	7.01	13.25	10.54	0	100	480
DBI	16.79	3.67	6.94	12.24	23.72	36.73	14.92	0	100	480
MHI	20.51	4.27	9.40	17.10	28.21	42.74	15.36	0	100	349
HHI	67.65	46.15	53.85	69.23	76.92	92.31	18.07	0	100	480
EAI	19.85	0.00	8.73	15.87	28.57	41.67	17.56	0	100	480

Descriptive statistics for the full sample.

### Treatment Effects and Estimation Methodologies

Having constructed the study indices, we next estimate the causal effects of (i) household displacement and (ii) residence in chars with Friendship NGO interventions on health and socio-economic outcomes. Because neither displacement nor NGO program placement is randomized, simple comparisons may be confounded by systematic differences between treated and control groups. We therefore adopt a potential-outcomes framework and estimate Average Treatment Effects (ATEs) under a selection-on-observables assumption, using multiple complementary estimators (regression adjustment, inverse-probability weighting, doubly robust approaches, and matching). We also assess covariate balance and robustness to support the credibility of the causal interpretation.

### Average Treatment Effect (ATE) Framework

To analyze the impact of displacement and Friendship NGO interventions, we employ the causal inference framework of [16] and define the Average Treatment Effect (ATE). For each household i , we define two potential outcomes, where $y_{0}\left(i\right)$ is the outcome if the household is not treated (e.g., non-displaced or residing in a control char), while $y_{1}\left(i\right)$ represents the outcome if the household is treated (e.g., displaced or residing in a treatment char).

In Model 1 (Equation 9), $y_{0}\left(i\right)$ represents the Disease Burden Index (DBI) for a non-displaced household, while $y_{1}\left(i\right)$ represents the DBI for a displaced household. In Model 2 (Equation 10), $y_{0}\left(i\right)$ and $y_{1}\left(i\right)$ correspond to the Migration Household Index (MHI) for households in control and treatment chars, respectively, allowing us to evaluate the impact of NGO interventions on migration dynamics.

The individual treatment effect and the Average Treatment Effect are defined in Equations 6, 7, with the sample estimator given in Equation 8.

$$\tau \left(i\right)=y_{1}\left(i\right)-y_{0}\left(i\right)$$ (6)

Since τ(i) varies across households, the ATE is defined as:

$$\text{ATE}=E\left[y_{1}-y_{0}\right]$$ (7)

which represents the average impact of displacement or NGO interventions on the outcome variable across all households.

Assuming ignorability of treatment assignment and a sufficiently large sample size, the ATE is estimated as:

$$\hat{\text{ATE}}=\frac{1}{N}\overset{N}{\underset{i=1}{\sum }}\left(y_{1}\left(i\right)-y_{0}\left(i\right)\right)$$ (8)

where N is the total number of households in the sample.

This framework enables robust estimation of causal effects, providing reliable insights into the socio-economic and health challenges faced by vulnerable populations in the chars of Bangladesh.

### Estimation Methodologies

To ensure robustness in estimating treatment effects, we employ several causal inference techniques. In this subsection we briefly describe, in intuitive terms, what each method does; more technical details and formal notation are provided in Supplementary Appendix E for interested readers.

1. Regression Adjustment (RA): Controls for confounding variables by adjusting for covariates in regression models [22].
2. Inverse Probability Weighting (IPW): Uses propensity scores to reweight observations, creating a pseudo-population where treatment assignment is independent of covariates [23].
3. Augmented Inverse Probability Weighting (AIPW): Combines outcome regression with IPW, yielding consistent estimates even if one component is misspecified [24].
4. Inverse Probability Weighted Regression Adjustment (IPWRA): Integrates IPW with regression adjustment for improved precision [25].
5. Nearest Neighbor Matching (NN): Pairs treated and control units based on covariates to enable direct comparisons between closely matched units [26].

These methodologies collectively form a robust framework for causal inference, allowing us to draw reliable conclusions about the effects of displacement and NGO interventions in the chars of Northeast Bangladesh. Further methodological details are provided in Supplementary Appendix E.

### Identification Assumptions and Covariate Balance

The causal interpretation of the estimated treatment effects relies on the standard identification assumptions used in the program-evaluation literature [16, 20, 21]. For both Model 1 and Model 2 we assume conditional ignorability, i.e.,

$$\left(y_{0}\left(i\right),y_{1}\left(i\right)\right)⊥D_{i}|X_{i},$$

where $D_{i}$ denotes the treatment indicator (displacement status in Model 1, treatment char in Model 2) and $X_{i}$ is the vector of observed covariates used in the propensity-score and outcome models. In our application, $X_{i}$ includes the Wealth Household Index (WHI), Hygiene Household Index (HHI), Educational Attainment Index (EAI), age of the respondent, agricultural and non-agricultural income quintiles, and fixed effects for chars. Intuitively, this assumption requires that, conditional on these covariates, treatment assignment is as good as random.

We also assume positivity (or overlap): for all values of $X_{i}$ in the support of the data, the probability of treatment is strictly between zero and one, so that each type of household has a non-zero chance of being both treated and untreated. This assumption is crucial for weighting and matching estimators, which rely on comparisons between treated and control observations with similar propensity scores. To assess overlap, Supplementary Appendix E reports the distributions of estimated propensity scores for treated and control groups in both models; these figures show substantial common support and no extreme concentrations near 0 or 1.

To evaluate whether conditioning on $X_{i}$ achieves adequate balance, we compute standardized mean differences (SMDs) for all covariates before and after applying the IPW, AIPW and IPWRA weights and after nearest-neighbour matching. Supplementary Table E.1 summarises these diagnostics. Prior to adjustment, some covariates exhibit absolute SMDs above commonly used thresholds (e.g., 0.2), indicating important differences between treated and control households. After weighting or matching, absolute SMDs for all main covariates are reduced to well below 0.1 in both models, which is typically viewed as evidence of good balance. These diagnostics, together with the propensity-score overlap plots, support the plausibility of our identification strategy, while we acknowledge that residual bias from unmeasured confounders cannot be ruled out in a cross-sectional design.

### Model Specifications

We estimate treatment effects using two models, each addressing a distinct research question. Model 1 (Equation 9) estimates the relationship between displacement and health outcomes (DBI). Model 2 (Equation 10) evaluates the impact of residing in treatment chars on migration dynamics (MHI). Both models account for potential biases and confounding factors [20, 21].

#### Model 1: Displacement and Disease Burden Index (DBI)

Model 1, Equation 9, examines the relationship between displacement and health outcomes, measured by the Disease Burden Index (DBI). Specifically, it estimates whether displaced households experience a higher disease burden compared to non-displaced households. The model is specified as:

$$P\left({\text{Displacement}}_{i}=1|DBI_{i}\right)=F\left(\alpha +\beta \cdot DBI_{i}+\gamma \cdot X_{i}\right),$$ (9)

where: $P\left({\text{Displacement}}_{i}=1\right)$ is the probability that household i is displaced, $DBI_{i}$ is the Disease Burden Index for household i , $X_{i}$ is a vector of socio-economic covariates, including the Wealth Household Index (WHI), Hygiene Household Index (HHI), and Educational Attainment Index (EAI). Additional controls include respondent age, agricultural and non-agricultural income quintiles, and fixed effects for chars to account for geographic heterogeneity. This model assesses whether poor health conditions significantly influence displacement likelihood.

#### Model 2: Treatment Char and Migration Household Index (MHI)

Model 2, Equation 10, evaluates how residing in a treatment char, where Friendship NGO operates, is associated with migration dynamics as captured by the Migration Household Index (MHI). As defined in Section *Methods*, higher values of MHI correspond to more intense or frequent migration events, while lower values indicate greater residential stability. Rather than assuming that NGO activities necessarily reduce migration, the model is designed to assess whether MHI systematically differs between treatment and control chars, conditional on observed covariates. The model is specified as:

$$P\left({\text{Treatment}}_{i}=1|MHI_{i}\right)=F\left(\alpha +\beta \cdot MHI_{i}+\gamma \cdot X_{i}\right),$$ (10)

where $P\left({\text{Treatment}}_{i}=1\right)$ is the probability that household i resides in a treatment char, $MHI_{i}$ is the Migration Household Index for household i , $X_{i}$ includes the Wealth Household Index (WHI), Hygiene Household Index (HHI), and Educational Attainment Index (EAI). Additional controls include respondent age, agricultural and non-agricultural income quintiles, and fixed effects for chars. This specification allows us to study how migration patterns vary between chars with and without Friendship interventions, while recognising that programme placement may itself be targeted towards areas experiencing stronger migration pressures. As a result, the estimated associations should be interpreted with caution and are not, by themselves, definitive estimates of the causal effect of NGO interventions on migration.

These models provide a structured approach to analyzing the relationship between displacement, health outcomes, and migration. Model 1, Equation 9, evaluates whether poor health conditions drive displacement, while Model 2, Equation 10, assesses the impact of NGO interventions on migration dynamics. Together, they offer insights into the socio-economic and health challenges faced by households in the chars of Bangladesh.

## Results

### Model 1: Disease Burden Index (DBI) of Displaced vs. Non-Displaced Peoples

Model 1, Equation 9, estimates the Average Treatment Effect (ATE) of displacement–measured by the binary variable Displ-NonDispl–on the Disease Burden Index (DBI), while controlling for a series of covariates, including observable char-specific characteristics to capture inter-Char variation.

The estimated ATE presented in Table 4 is significantly negative, ranging from −4.85 to −6.19 , indicating that, when controlling for covariates, displaced households report a substantial reduction in disease burden compared to non-displaced households. This pattern is counterintuitive in light of the broader literature, yet it is consistent with a combination of mechanisms. One possibility is that displaced households, particularly in treatment chars, have better access to Friendship NGO services or other providers (for example, through mobile clinics or health outreach), which may improve prevention, diagnosis and treatment and thus reduce their disease burden. Another possibility is that displacement changes health-seeking behaviour and the way symptoms are perceived and reported, leading to systematic differences in how illness episodes are recorded in the survey. Finally, selection mechanisms, including forms of “survivor bias”, in which healthier, more resilient households are more likely to remain observable in the displaced population, may also shape which households we observe in this cross-sectional sample.

**TABLE 4:** Average treatment effects (ATE) of displacement on the disease burden index (DBI), model 1: Bangladesh, 2022 (Equation 9) (Friendship NGO household survey, Bangladesh, 2022).

Statistic	(1)	(2)	(3)	(4)	(5)
	RA	IPW	AIPW	IPWRA	NN
ATE	−4.85**	−4.20**	−4.19**	−4.21**	−6.19**
​	(2.04)	(1.95)	(1.95)	(1.85)	(2.54)
POmean	24.06***	23.34***	23.36***	23.37***	​
​	(1.85)	(1.75)	(1.73)	(1.64)	​
Observations (N)	241	241	241	241	241

Standard errors in parentheses. Significance level: * p<0.10 , ** p<0.05 , *** p<0.01 . DBI is scaled from 0 to 100, with higher values indicating worse health. The ATE row reports the estimated average difference in DBI (in index points) between displaced (treated) and non-displaced (control) households. Negative values therefore imply a lower disease burden among displaced households. POmean gives the expected DBI for non-displaced households (control group).

The Potential Outcome Mean (POmean) for non-displaced individuals–ranging from 23.34 to 24.06 – indicates a consistently high disease burden in more stable habitats, likely exacerbated by systemic health and sanitation challenges and, in some cases, more limited contact with NGO services. Taken together, these results suggest that the lower DBI observed among displaced households should not be interpreted as evidence that displacement is benign, but rather that the combination of service access, reporting differences and selection may partially mask underlying health risks among the most vulnerable.

Because we observe households at a single point in time, we cannot empirically disentangle these mechanisms. In Section *Discussion* we therefore treat survivor bias and other forms of selection as plausible, but not definitive, explanations for the lower DBI among displaced households.

Table 4 summarizes Model 1 (Equation 9) treatment effect estimations:

The estimates in Table 4 indicate that, after adjusting for covariates, displaced households have on average between 4 and 6 index points lower DBI than comparable non-displaced households. Given that the DBI ranges from 0 to 100 and the expected DBI for the non-displaced group (POmean) is around 23–24 points, this corresponds to roughly a 20%–25% reduction in the average disease burden among displaced households relative to non-displaced ones in our sample. This magnitude helps quantify the practical importance of the negative ATE, beyond its statistical significance.

While displacement typically heightens vulnerability, these findings suggest that targeted interventions can mitigate health impacts, even though non-displaced groups still face significant risks from limited healthcare and sanitation.

### Model 2: Migration Households Index (MHI) of Friendship NGO’s Treated Chars

Model 2, Equation 10, estimates the Migration Household Index (MHI) as the outcome variable, representing the migration index, with the treatment variable identifying chars where Friendship NGO implements health and educational activities. This model incorporates the same covariates as Model 1.

Across all methodologies, the ATE estimate reported in Table 5 is significantly positive, ranging from 9.93 to 10.45. Given that higher values of MHI indicate more intense or frequent migration, these results imply that households in treatment chars exhibit, on average, higher migration intensity than comparable households in control chars, after conditioning on the covariates in the model. One plausible explanation is that Friendship tends to operate in chars already experiencing stronger migration pressures, so that treatment status partly reflects programme targeting rather than the causal impact of the intervention itself. We therefore interpret these estimates as evidence of systematic differences in migration patterns between treatment and control chars, rather than as demonstrating that NGO activities increase or decrease migration.

**TABLE 5:** Average treatment effects (ATE) of treatment char residence on the migration household index (MHI), model: Bangladesh, 2022 (Equation 10) (Friendship NGO household survey, Bangladesh, 2022).

Statistic	(1)	(2)	(3)	(4)	(5)
	RA	IPW	AIPW	IPWRA	NN
ATE	9.95***	10.12***	9.93***	10.05***	10.45***
​	(1.85)	(1.75)	(1.85)	(1.80)	(1.85)
POmean	8.69***	8.59***	8.76***	8.64***	​
​	(1.45)	(1.32)	(1.45)	(1.39)	​
Observations (N)	240	240	240	240	240

Standard errors in parentheses. Significance level: * p<0.10 , ** p<0.05 , *** p<0.01 . MHI is scaled from 0 to 100, with higher values indicating more intense or frequent migration. The ATE row reports the estimated average difference in MHI (in index points) between households in treatment chars (with Friendship NGO interventions) and control chars. Positive values therefore imply higher migration intensity in treatment chars. POmean gives the expected MHI for households in control chars.

The Potential Outcome Mean (POmean), also shown in Table 5, represents the expected migration index for the control group. It is consistently high (ranging from 8.59 to 8.76), which points to a non-trivial baseline level of migration activity even among households in chars without Friendship interventions and reflects pre-existing migration trends and pressures in the study area.

Table 5 summarizes Model 2 (Equation 10) treatment effect estimations:

From Table 5, the estimated ATEs of roughly 10 index points imply that households living in chars where Friendship operates have, on average, substantially higher migration intensity than similar households in control chars, conditional on the covariates in the model. Since the MHI also ranges from 0 to 100 and the expected value for the control group (POmean) is about 9 points, the positive ATE indicates that treatment-char households experience migration levels that are roughly double those of households in non-program chars. This interpretation makes the scale and direction of the MHI differences easier to relate to concrete migration pressures.

### Robustness Checks

To ensure the robustness of our estimations, we performed a Variance Inflation Factor (VIF) analysis to examine potential multicollinearity among the constructed indexes; the results are presented in Supplementary Appendix D. Additionally, we computed the ROC curve and AUC for each model to evaluate their discriminatory performance. The following section presents the Receiver Operating Characteristic (ROC) curve.

#### Receiver Operating Characteristic (ROC) Curve

The area under the Receiver Operating Characteristic (ROC) curve (AUC) was utilized to assess the predictive performance and overall quality of the model. The ROC curve is a widely used tool for evaluating the performance of binary classification models [28, 29]. The AUC measures the model’s ability to distinguish between positive and negative outcomes, with a value closer to 1 indicating excellent discriminative capability and a value of 0.5 suggesting no discrimination [30]. This analysis assessed the models’ ability to predict outcomes such as displacement status or access to interventions based on the explanatory variables. For instance, the ROC curve for the logistic regression model analyzing the Disease Burden Index (DBI) in Model 1 Equation 9 yielded an AUC of 0.81, indicating an excellent discriminatory power. Similarly, the ROC curves for Model 2 Equation 10, those estimating migration patterns, demonstrated good performance levels, further supporting the validity of the causal inferences drawn from the analysis.

#### Model 1 (Disease Burden Index and Displacement Status): ROC Curve

**FIGURE 1:**
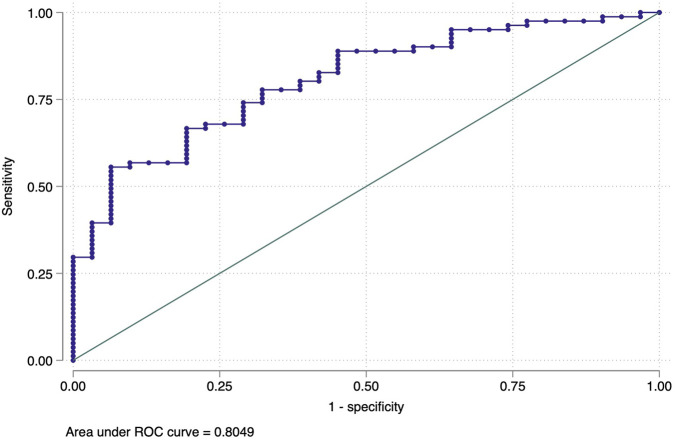
The receiver operating characteristic curve of Model 1 (Disease Burden Index and Displacement Status: Bangladesh, 2022). Notes: The receiver operating characteristic curve visually represents model performance by plotting the true positive rate (sensitivity) against the false positive rate (1-specificity) across various classification thresholds. The Area Under the Curve (AUC) serves as a summary statistic, quantifying the model’s ability to discriminate between classes. An AUC of 0.5 indicates no discriminatory power (equivalent to random guessing), while an AUC of 1.0 signifies perfect classification (Friendship NGO household survey, Bangladesh, 2022).

The ROC curve in Figure 1 illustrates the performance of the logistic regression model analyzing the Disease Burden Index (DBI) by displacement status (Model 1, Equation 9). With an AUC of 0.8049, the model demonstrates strong discriminatory power, effectively distinguishing between displaced and non-displaced households.

#### Model 2 (Migration Index and NGO Interventions): ROC Curve

**FIGURE 2:**
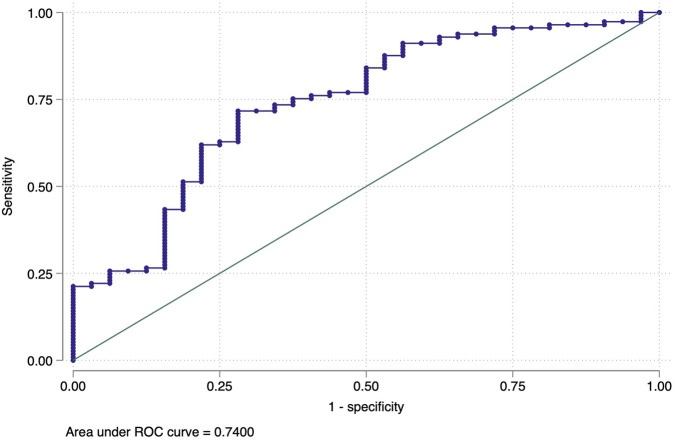
The receiver operating characteristic curve of Model 2 (Migration Index and NGO Interventions: Bangladesh, 2022). Notes: The receiver operating characteristic curve visually represents model performance by plotting the true positive rate (sensitivity) against the false positive rate (1-specificity) across various classification thresholds. The Area Under the Curve (AUC) serves as a summary statistic, quantifying the model’s ability to discriminate between classes. An AUC of 0.5 indicates no discriminatory power (equivalent to random guessing), while an AUC of 1.0 signifies perfect classification (Friendship NGO household survey, Bangladesh, 2022).

The ROC curve in Figure 2 evaluates the logistic regression model analyzing the Migration Household Index (MHI) by treatment status (Friendship NGO intervention) (Model 2, Equation 10). An AUC of 0.74 indicates good discriminatory power, demonstrating the model’s effectiveness in distinguishing between treated and control chars.

### Results Interpretation

Displaced households show significantly lower DBI scores than non-displaced ones, even after adjusting for wealth, hygiene, educational attainment, age, income quintiles and char fixed effects. At face value, this pattern is consistent with beneficial effects of targeted health interventions and improved access to services in some displaced settings, as well as with effective coping strategies that reduce the burden of illness among households that are able to relocate.

At the same time, this finding is counterintuitive given the broader literature documenting that displacement typically heightens health risks. Several, non-mutually-exclusive mechanisms may help explain the result. First, treatment chars are precisely those where Friendship NGO operates, so displaced households in these areas may enjoy better access to preventive and curative care than non-displaced households in less-served locations. Second, displacement may influence how symptoms are perceived, normalised or reported, generating potential reporting and recall differences between displaced and non-displaced groups. Third, selection mechanisms–including forms of “survivor bias” in which more resilient households are more likely to remain observable among the displaced–could lead the most vulnerable households to exit the sampling frame through severe morbidity, mortality or onward migration.

Because our data are cross-sectional, we cannot empirically separate these mechanisms, and we therefore present survivor bias and related selection processes as plausible rather than definitive explanations. The robustness checks and sensitivity analysis reported in Supplementary Appendix E help bound the extent to which unmeasured selection would need to operate to overturn our main findings and highlight the need for future longitudinal work that follows households over time.

## Discussion

Displaced households show significantly lower DBI scores than non-displaced ones, even after adjusting for wealth, hygiene, educational attainment, age, income quintiles and char fixed effects. At face value, this pattern is consistent with beneficial effects of targeted health interventions and improved access to services in some displaced settings, as well as with effective coping strategies that reduce the burden of illness among households that are able to relocate. At the same time, this finding is counterintuitive given the broader literature documenting that displacement typically heightens health risks. Several, non-mutually-exclusive mechanisms may help explain the result. First, treatment chars are precisely those where Friendship NGO operates, so displaced households in these areas may enjoy better access to preventive and curative care than non-displaced households in less-served locations. Second, displacement may influence how symptoms are perceived, normalised or reported, generating potential reporting and recall differences between displaced and non-displaced groups. Third, selection mechanisms–including forms of “survivor bias” in which more resilient households are more likely to remain observable among the displaced–could lead the most vulnerable households to exit the sampling frame through severe morbidity, mortality or onward migration. Because our data are cross-sectional, we cannot empirically separate these mechanisms, and we therefore present survivor bias and related selection processes as plausible rather than definitive explanations. The robustness checks and sensitivity analysis reported in Supplementary Appendix E help bound the extent to which unmeasured selection would need to operate to overturn our main findings and highlight the need for future longitudinal work that follows households over time.

### Limitations of the Study

This study provides valuable insights into the health, migration, and socio-economic conditions of char households in North-East Bangladesh, but it has several important limitations. First, our analysis is based on a single cross-sectional survey conducted between March and June 2022. As a result, the data provide a snapshot rather than a dynamic picture, and we cannot observe pre-displacement baselines, post-displacement adaptation, or long-run trajectories of health and migration. The causal interpretation of the estimated treatment effects is therefore confined to this cross-sectional setting, under the stated identification assumptions, and does not extend to changes over time.

Second, all outcome variables are self-reported, which may introduce recall error and reporting bias. Differences in how displaced and non-displaced households perceive and report symptoms, income, or migration events could affect the constructed indices, including the Disease Burden Index (DBI) and Migration Household Index (MHI). Although we attempt to mitigate these issues through careful questionnaire design and index construction, we cannot fully rule out systematic reporting differences.

Third, while the quasi-experimental estimators adjust for a rich set of observed covariates, they cannot eliminate bias from unmeasured historical, environmental, or socio-economic factors. We cannot directly observe households that may have dissolved, experienced mortality, or migrated out of the study area, so selection mechanisms—including possible forms of “survivor bias”— remain a concern. As a consequence, our estimates should be interpreted as associations that are consistent with causal effects under the conditional ignorability assumption, rather than as definitive causal effects that hold under all forms of unobserved heterogeneity.

Fourth, the implications for external validity are limited by the specific context of the chars and by our sampling frame. The studied chars are riverine islands with highly dynamic landforms, recurrent erosion, and particular settlement and livelihood patterns, and the treatment chars host long-standing Friendship NGO health and education programmes. These physical and institutional conditions differ from other displacement settings in South Asia, such as coastal deltas, urban informal settlements, or conflict-affected areas, where both hazards and service provision may look very different. In addition, the survey interviews one primary respondent per household, restricted to women aged 15–55, which reflects Friendship NGO’s programmatic focus and the fact that women in this age group are typically the main caregivers and best informed about health, hygiene and education decisions. Households without an eligible woman (for example, male-only or elderly-only households) are therefore not represented. Our quantitative estimates are thus most directly generalisable to similar char households in North-East Bangladesh, rather than to all displacement-prone contexts in South Asia.

Despite these drawbacks, the study lays a foundation for understanding the links between temporary displacement, health and socio-economic vulnerability in Bangladesh’s chars. It also highlights the need for longitudinal and multi-site research that follows households over time and compares different types of displacement-prone environments across South Asia.

### Conclusion

Our study uses a cross-sectional quasi-experimental framework and a set of household indices (DBI, MHI, WHI, HHI, EAI) to examine how temporary displacement and NGO presence are associated with health and socio-economic outcomes in chars in North-East Bangladesh. Within this setting and under the stated identification assumptions, the estimated treatment effects provide evidence on how displacement status and residence in treatment chars relate to disease burden and migration dynamics at the time of the survey.

For health outcomes, we find that displaced households in areas with Friendship NGO interventions exhibit lower DBI scores than comparable non-displaced households. This pattern is consistent with targeted health measures and better access to services helping to reduce disease burden among displaced households, but may also reflect differences in reporting behaviour and selection in which households remain observable. For migration dynamics, chars where Friendship operates show systematically different—and, on average, higher—MHI scores than control chars, which likely reflects both underlying migration pressures and the targeted placement of programmes. Because our data are cross-sectional and programme placement is not random, these associations should not be interpreted as definitive evidence that NGO activities increase or decrease migration over time.

More broadly, our results reveal distinct patterns of migration, economic resources, hygiene and education across displaced and non-displaced households and between chars with and without NGO interventions. Conceptually, the integrated use of health, migration and socio-economic indices illustrates how different dimensions of vulnerability and resilience interact in a climate-vulnerable riverine environment. However, the quantitative estimates are directly applicable to the char context studied here and to similar settings with comparable hazards and programmatic conditions; they should be extrapolated to other displacement-prone areas in South Asia only with caution and appropriate empirical validation.

Future research should build on this work by collecting longitudinal data that track the same households over time and by comparing multiple types of displacement-prone environments across South Asia. Such studies would allow a more precise estimation of dynamic causal effects of displacement and interventions on health and migration, and would help to design adaptive strategies that are tailored to diverse local contexts. Within these limitations, our findings provide useful guidance for policymakers and practitioners seeking to reduce disease burden and support household resilience in Bangladesh’s chars.
