# Supplementary material for: Health and Socio-Economic Impacts of Climate-Related Displacement in Bangladesh’s Chars: Causal Evidence From a Household Survey
Source: Int J Public Health. 2026 Mar 11;71:1608475. doi: 10.3389/ijph.2026.1608475 (PMC13013082; doi:10.3389/ijph.2026.1608475)
Supplement: Supplementary file 1 [file DataSheet1.pdf]

Supplementary Appendix

Health and Socio-Economic Impacts of Climate-Related Displacement in Bangladesh's Chars: Causal Evidence from a Household Survey

Juan A. de Castro[a] and Laurentiu Guinea[b*]

[a]Universidad Nebrija, Madrid
[b]Universidad Complutense de Madrid and ICAE

December 2025

Abstract

Objectives: To assess health and socio-economic impacts of climate-related displacement in North-East Bangladesh chars and examine links between non-governmental services, disease burden and migration.

Methods: We analysed a household survey of 480 women aged 15–55 from nine intervention and three comparison chars, collected between March and June 2022. Using a quasi-experimental framework and estimators of the average treatment effect, we compared displaced and non-displaced households and households in chars with and without Friendship health and education services. We constructed indices of disease burden, migration and socio-economic conditions, each scaled 0–100.

Results: Displaced households had lower disease burden scores than non-displaced households after adjusting for socio-economic covariates. This pattern is consistent with improved access to services among some displaced groups, but may also reflect reporting differences and selection into the observed displaced population. Migration intensity was higher in chars where Friendship operates than in comparison chars, suggesting programme placement in areas with stronger migration pressures.

Conclusion: Climate-related displacement interacts with service access, vulnerability and selection in complex ways; targeted interventions can reduce disease burden but do not necessarily lower migration pressures.

JEL Classification: C21, C93, I12, I15, O15, Q54
Keywords: Causal Inference, Treatment Effects, Displacement, Disease Burden, Migration, Economic Vulnerability, Household Resilience.

*Corresponding Author: Laurentiu Guinea, ICAE, Universidad Complutense de Madrid, 28223 Madrid; E-mail: lguinea@ucm.es

Appendix

A Survey Design

Table A.1: Observations by Char and Accumulated Distribution

Char Name	Observations	Percent (%)	Cumulative (%)
BAJRA DIYAR KAHTA (T)	40	8.33	8.33
BATIKAMARI (T)	40	8.33	16.67
CHAR JATRAPUR (T)	40	8.33	25.00
CHOR GARUHARA (C)	40	8.33	33.33
DATIAR CHAR (C)	40	8.33	41.67
KAWA BADA (T)	40	8.33	50.00
KHAMAR BASHPATA (T)	40	8.33	58.33
KHEYAR ALGA (T)	40	8.33	66.67
KORAI BARISHAL (T)	40	8.33	75.00
KHAMAR HOLOKHANA (T)	40	8.33	83.33
MOHESHBANDI (C)	40	8.33	91.67
SOUTH SANNASIR CHAR (T)	40	8.33	100.00
**Total**	**480**	**100.00**	**100.00**

Note: (T) indicates treated chars and (C) indicates control chars.

A.1 Respondent Selection

The survey employed a stratified random sampling approach to ensure representativeness across treatment and control chars. "Treatment chars" were defined as those where Friendship NGO actively implements health and education interventions, while "control chars" lacked such programs.

Within each char, 40 households were randomly selected for participation. The final sample comprised 480 households, distributed across 12 chars (nine treatment and three control chars).

A.2 Survey Respondents

The survey respondents were women, providing insights that accurately captured household-level dynamics. To ensure data reliability, enumerators underwent training to conduct follow-up questions and perform consistency checks, enhancing the accuracy and credibility of the collected information.

A.3 Questionnaire Design

The questionnaire was designed to collect comprehensive data across six key dimensions as follows:

i) The demographic information gathered details about the age, gender, education, and occupation of household members, providing a foundational understanding of the surveyed population.

ii) The health conditions where respondents reported illnesses experienced by household members over the past six months, including diseases such as diarrhea, typhoid, and skin infections and specific questions addressed whether household members had been affected by infectious diseases, including Typhoid, Hepatitis (A, B, C), Cholera, Diarrhea, Worms, Dysentery, and Covid-19. This data was crucial for constructing the Disease Burden Index (DBI).

iii) The migration patterns collected information on temporary or permanent displacement, including the number of days displaced, the frequency of migration, and the reasons for migration (e.g., river erosion, urbanization, poverty). This data informed the construction of the Migration Household Index (MHI).

iv) The socio-economic status collected data on household income from agriculture and non-agriculture activities, land ownership, livestock, and assets. This information was used to construct the Wealth Household Index (WHI).

v) The hygiene practices and infrastructure section assessed access to sanitation facilities, water sources, and hygiene-related behaviors, forming the basis for the Hygiene Household Index (HHI).

vi) The educational attainment reported data about the level of education achieved by household

members. This information was used to compute the Educational Attainment Index (EAI).

A.4 Survey Implementation and Ethical Considerations

The survey was conducted between March, 2022 and June, 2022. A team of trained enumerators carried out face-to-face interviews using structured questionnaires. Data was collected using both paper-based and digital formats to ensure accuracy and facilitate timely processing. Enumerators underwent a rigorous training program to ensure they were familiar with the questionnaire and understood the importance of maintaining neutrality and cultural sensitivity during interviews.

The survey followed strict ethical guidelines to protect respondents' privacy and confidentiality. Participation was voluntary, and informed consent was obtained from all respondents prior to the interview. Respondents were assured that their data would be anonymized and used solely for research purposes.

B Char migration factors

The data presented in Table B.1 highlights the primary factors driving migration in the chars of North-East Bangladesh. Among these factors, river erosion, urbanization, and poverty-induced relocation to Dhaka emerge as the most significant, followed by landlessness, family conflict, and other causes. This distribution of migration illustrates the environmental, economic, and social pressures that drive households to relocate.

River erosion (Table B.1) is identified as the leading cause of migration, reflecting the direct impact of climate change on the chars. The increasing frequency and severity of flooding events, driven by changing weather patterns, erode arable land, destroy homes, and disrupt livelihoods. For households heavily reliant on agriculture and natural resources, the loss of land and infrastructure compels them to temporarily migrate in pursuit of family safety and economic stability. River erosion exemplifies how environmental degradation directly displaces communities, creating a cycle of vulnerability and migration.

Urbanization, (Table B.1) the second most important factor, reflects a dual dynamic: it serves as both a response to and a consequence of climate change. Many households migrate to urban centers

seeking better economic opportunities, healthcare, and education, driven in part by the decline in agricultural productivity caused by climate-induced loss of arable land. As river erosion and other environmental shocks render farming unsustainable, urban areas become an attractive alternative for displaced populations. This pattern highlights the growing disparities between rural and urban regions, where climate change intensifies rural poverty and migration exacerbates urban challenges like overcrowding and limited infrastructure.

Poverty-induced relocation to Dhaka emerges as another key driver of migration, closely linked to economic distress (Table B.1). Many households face limited access to sustainable livelihoods in the chars, and repeated environmental shocks such as river flooding exacerbate their financial instability. The absence of robust social safety nets further compounds their vulnerability, compelling them to migrate to Dhaka in search of income and security.

Landlessness also plays a significant role, particularly in contexts where agricultural productivity is central to livelihoods (Table B.1). Households without secure land tenure are more susceptible to displacement, as they lack the resources needed to recover from environmental shocks. This reinforces the structural inequalities that perpetuate cycles of vulnerability and migration in the chars.

Family conflict and other causes, while less significant in the aggregate, nonetheless highlight the diverse and multifaceted reasons behind migration. Family disputes can disrupt household cohesion, leading to relocation, while "other causes" may include a range of personal, social, and cultural factors not captured by the primary categories.

In conclusion, the causes of migration in the chars reflect a combination of environmental, economic, and social pressures, with river erosion, urbanization, and poverty standing out as the most critical drivers. Notably, urbanization, as an indirect response to climate-induced loss of agricultural land in a region with a strong agricultural tradition, highlights between environmental degradation and socio-economic transformation. Addressing these challenges requires a comprehensive approach that integrates climate adaptation, rural development, and urban planning. Investments in flood management infrastructure, sustainable livelihoods, and targeted social protection programs can mitigate the adverse impacts of river erosion and poverty, while balanced urban development policies can support rural migrants in achieving a better quality of life.

Table B.1: Reasons for Migration by Char Name

Char Name	River Erosion	Urbanization	Landless	Family Conflict	Moved to Dhaka due to Poverty	Not Found	Total
BAJRA DIYAR KHATA (T)	15	6	3	0	0	0	24
BATIKAMARI (T)	18	5	0	0	0	0	23
CHAR JATRAPUR (T)	0	0	0	1	3	0	4
CHOR GARUHARA (C)	12	0	0	0	6	4	22
DATIAR CHAR (C)	23	0	0	0	0	0	23
KAWA BADA (T)	32	5	0	0	0	0	37
KHAMAR BASHPATA (T)	9	0	0	0	0	0	9
KHAMAR HOLOKHANA (T)	0	0	3	0	0	0	3
KHEYAR ALGA (T)	17	0	0	0	0	0	17
KORAI BARISHAL (T)	8	4	0	0	0	0	12
MOHESHBANDI (C)	13	6	3	0	0	0	22
SOUTH SANNASIR CHAR (T)	16	0	0	0	0	0	16
**Total**	**163**	**26**	**9**	**1**	**9**	**4**	**212**

C Methodology for Index Construction

The construction of the indices used in this study quantify and analyze various socio-economic and health dimensions. Each index was designed to reflect specific aspects of household conditions, providing a framework for evaluating the impacts of climate-induced displacement and targeted interventions. The indices include the Disease Burden Index (DBI), Migration Household Index (MHI), Wealth Household Index (WHI), Hygiene Household Index (HHI), and Educational Attainment Index (EAI). Below, we outline the methodology applied for the construction.

C.1 Migration Household Index (MHI)

The Migration Household Index (MHI) is designed to quantify the extent and dynamics of migration experienced by households. This index captures both the frequency and type of migration, providing

a comprehensive measure to analyze the socio-economic and environmental factors driving household mobility, particularly in response to climate-induced challenges.

The construction of the MHI is based on survey data that records migration-related information for each household, including: whether migrated or not, the frequency of migration, the number of times a household or its members have migrated and the average numbers of days each time have migrated and reasons for migration, factors such as climate-related events (e.g., flooding, erosion), economic opportunities, or social circumstances.

C.1.1 Calculation of MHI

The Migration Household Index is constructed using the following formula:

$$\text{MHI} = \frac{\sum_{i=1}^{N} \left(\text{Weight}_{\text{type},i} \times \text{Frequency}_i \times \text{Proportion}_{\text{members},i}\right)}{\text{Total Household Members}}, \tag{C.1}$$

where: N represents the total number of migration events reported by the household, $\text{Weight}_{\text{type},i}$ represents the weight assigned to each type of migration based on its socio-economic impact (e.g., permanent migration may carry a higher weight than seasonal migration), Frequency_i represents the number of times a specific type of migration occurred $\text{Proportion}_{\text{members},i}$: The proportion of household members involved in each migration event. Total Household Members: Total number of individuals in the household.

The raw MHI is then normalized to ensure comparability across households:

$$\text{Normalized MHI} = \frac{\text{Raw MHI} - \text{Minimum MHI}}{\text{Maximum MHI} - \text{Minimum MHI}} \times 100, \tag{C.2}$$

where *Minimum MHI* and *Maximum MHI* represent the observed minimum and maximum values of the raw index in the dataset.

C.1.2 Interpretation of MHI

The MHI provides a standardized measure of household migration dynamics:

- A higher MHI indicates a household with frequent and/or impactful migration, suggesting

greater mobility due to socio-economic or environmental factors.

- A lower MHI reflects minimal migration activity, potentially indicating stability or limited access to adaptive opportunities.

C.1.3 Relevance of MHI in the Study

The MHI plays a crucial role in understanding the migration patterns within the chars of North-East Bangladesh, where households face recurrent displacement due to flooding and erosion. By analyzing the MHI, we can evaluate the impact of external interventions, such as those by Friendship NGO, on reducing forced migration and enhancing household resilience.

The MHI is also used to assess how migration contributes to coping mechanisms and adaptive strategies in vulnerable communities, offering insights for designing effective policies to mitigate the adverse effects of climate-induced displacement.

Histograms of the MHI are presented, disaggregated by treatment, Figure C.1. The histogram allows for a comparative analysis of migration dynamics between households residing in treatment chars (where Friendship NGO operates) and control chars.

Scatter plots of the MHI by age are generated, stratified by treatment, Figure C.2. These plots illustrate the relationship between household migration dynamics and age, highlighting variations in migration behavior across different demographic groups.

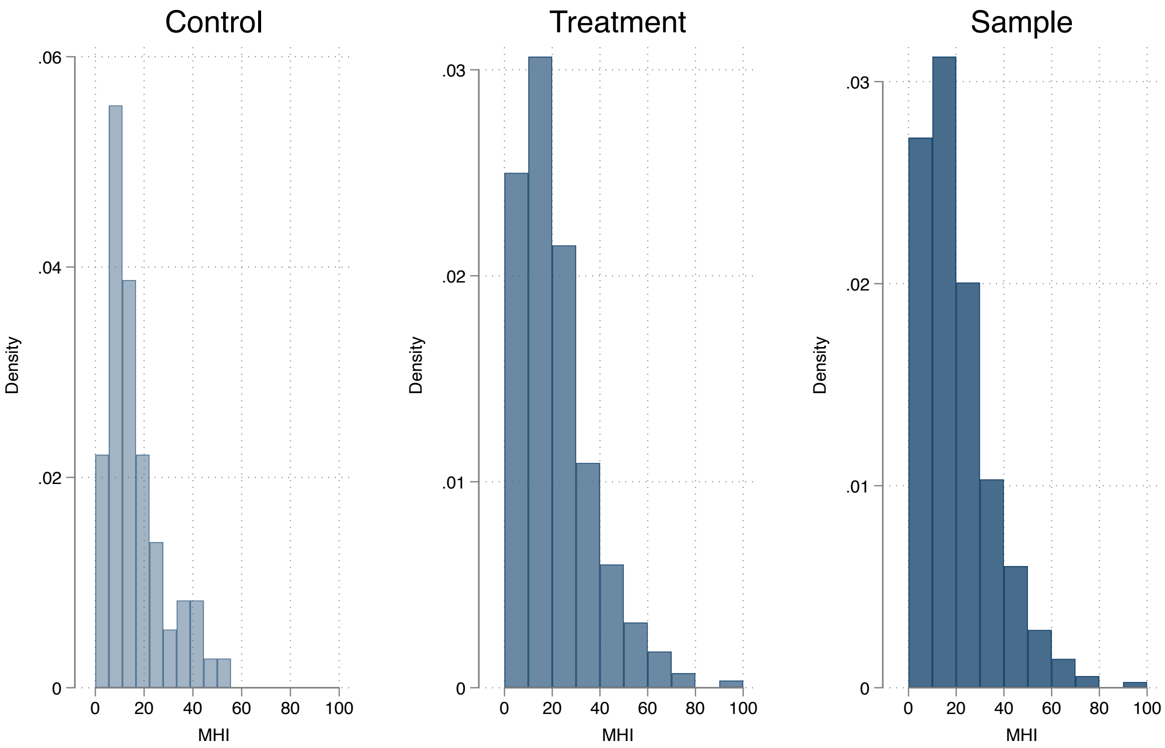

Figure C.1: Distribution of the **Migration Household Index (MHI)** for Treatment and Control Groups

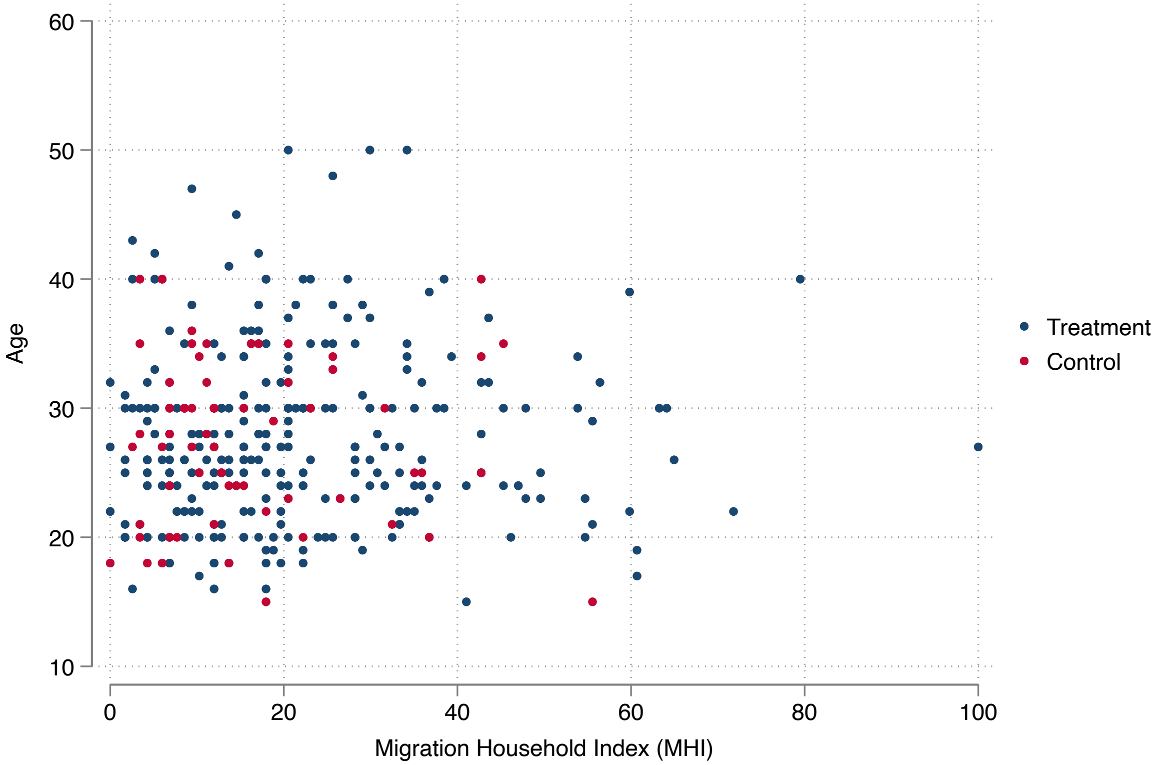

Figure C.2: Age Distribution against the Migration Household Index (MHI) for Treatment and Control Groups

C.2 Disease Burden Index (DBI)

The Disease Burden Index (DBI) was developed to quantify the prevalence and severity of health conditions reported by households in both treatment and control populations within the chars of North-East Bangladesh. By accounting for both the proportion of affected household members and the severity of reported illnesses, the DBI provides a comprehensive and standardized measure for assessing health impacts and evaluating vulnerabilities.

We follow **(author?)** (1) to construct the Disease Burden Index, DBI_i:

$$DBI_i = \frac{T_i}{N_i} \times 100, \tag{C.3}$$

where DBI_i is the index of illness for household i, which quantifies the impact or burden of illness on individuals. Numerator T_i represents the type of illness across all the household members. N_i

represents the total number of members present in household i. The index takes a positive value if at least one member reports illness at any given date and is zero if no one within the household reports illness. Eq. (C.3) captures both the proportion of ill members and the severity of illness amongst the household members. To see this, we define n_i to denote the number of types reporting illness in the household. If $n_i > 0$, then the index can be decomposed into into:

$$DBI_i = \frac{n_i}{N_i} \times \frac{T_i}{n_i} \times 100 = dbi_i^p \times dbi_i^s \times 100 \tag{C.4}$$

where dbi_i^p represents the proportion of household members suffering from illness and dbi_i^s captures the severity of the illness shock. Specifically, DBI_i represents the proportion of the health burden borne by each ill member due to the severity of their illness.

C.2.1 Weights and ICD-10 Integration in the DBI

We incorporate into the Disease Burden Index (DBI) multiple dimensions of health challenges, including the prevalence of specific diseases, the severity of symptoms, the duration of illness, and the resulting effects on daily functioning and quality of life. We start identifying from the questions "Did your family members suffer any diseases within last 6 month?" and "Are you or your family members affected by any infectious /communicable diseases?" the specific diseases or health conditions included in the index. These encompass a range of infectious and non-infectious diseases that are prevalent in the study population, such as malaria, diarrhea, typhoid, and Covid-19, among others. Each of these diseases is mapped to its corresponding International Classification of Diseases, 10th Revision (ICD-10) code to ensure consistency and comparability with global health standards. Weights are then assigned to each disease based on three primary factors. The first one is the prevalence: how common the disease is within the study population. The second one is the severity: the degree of disability, discomfort, or impairment caused by the disease. Finally we consider the impact on quality of life: the extent to which the disease disrupts daily activities, productivity, or overall well-being.

Diseases with higher prevalence rates or those associated with severe symptoms and significant impairments are assigned greater weights, reflecting their larger contribution to the overall disease burden. For example, acute conditions such as pneumonia or Covid-19, which are life-threatening

and have systemic impacts, receive higher weights compared to minor conditions like muscle soreness or skin diseases.

Once weights are assigned, the DBI for each individual or household is calculated by aggregating the weighted scores across all included diseases. This yields a single numerical value that reflects the overall burden of disease experienced by the population.

Table C.1 presents the list of diseases included in the DBI, along with their corresponding ICD-10 codes. This alignment with ICD-10 facilitates integration with broader health datasets and ensures the DBI aligns with international health classification standards.

Table C.1: Weights and ICD-10 Codes for All Diseases in the Study

**Disease**	**Severity/Impact Level**	**Weight**	**ICD-10 Code**
Diarrhea	Moderate (common, treatable)	3.5	A09
Blood Dysentery	High (serious, requires care)	4.5	A03
Typhoid	High (systemic, severe impact)	4.5	A01
ARI/Pneumonia	Very High (life-threatening)	5.5	J10–J18
Skin Diseases	Low (non-life-threatening)	2.0	L00–L99
Jaundice	High (can indicate liver damage)	3.5	R17
Fever and Cough	Moderate (common, mild)	1.5	J00–J06
Malaria	Very High (fatal if untreated)	4.5	B50–B54
Muscle Soreness	Low (minor health impact)	2.0	M79.1
Hepatitis (A, C, D)	High (liver-related, systemic impact)	4.0	B15, B16.0, B17.1
Cholera	High (acute, potentially fatal)	4.5	A00
Worms	Low (minor health impact)	2.0	B65–B83
Covid-19	Very High (pandemic, systemic impact)	5.0	U07.1
Others	Varies based on specific disease	0.5	Various

The DBI is calculated for each household by summing the weights of diseases reported in the survey. To ensure comparability, the DBI is normalized to range between 0 and 100 using the following formula:

$$\text{Normalized DBI} = \frac{\text{Raw DBI Value}}{\text{Maximum Possible DBI}} \times 100. \tag{C.5}$$

To standardize the index and enhance its comparability with global health metrics, these diseases were mapped to their corresponding ICD-10 (International Classification of Diseases, 10th Revision) codes,[1] as shown in Table C.2.

Table C.2: ICD-10 Codes for Diseases Included in the Study

**Disease**	**ICD-10 Code**
Diarrhea	A09 (Infectious gastroenteritis and colitis)
Blood Dysentery	A03 (Shigellosis)
Typhoid	A01 (Typhoid and paratyphoid fevers)
ARI/Pneumonia	J10–J18 (Influenza and pneumonia)
Skin Diseases	L00–L99 (Diseases of the skin and subcutaneous tissue)
Jaundice	R17 (Unspecified jaundice)
Fever and Cough	J00–J06 (Acute upper respiratory infections)
Malaria	B50?B54 (Malaria)
Muscle Soreness	M79.1 (Myalgia)
Hepatitis (A, C, D)	B15 (Hepatitis A), B16.0 (Hepatitis D), B17.1 (Hepatitis C)
Cholera	A00 (Cholera)
Worms	B65–B83 (Helminthiases)
Covid-19	U07.1 (COVID-19, virus identified)
Others	Various ICD-10 codes

To illustrate the distribution of health burdens across the surveyed population, histograms are presented for the DBI, disaggregated by treatment, Figure C.3 and displacement status, Figure C.4. These histograms allow for a clear comparison of health vulnerabilities between households in treatment chars (where Friendship NGO operates) and control chars, as well as between displaced and non-displaced households.

To further analyze the relationship between age and health outcomes, scatter plots of the DBI are presented, stratified by treatment, Figure C.5 and displacement status, Figure C.6. These plots provide insights into how age influences health burdens across different groups, capturing potential variations among younger and older household members.

[1] ICD-10 Classification Website

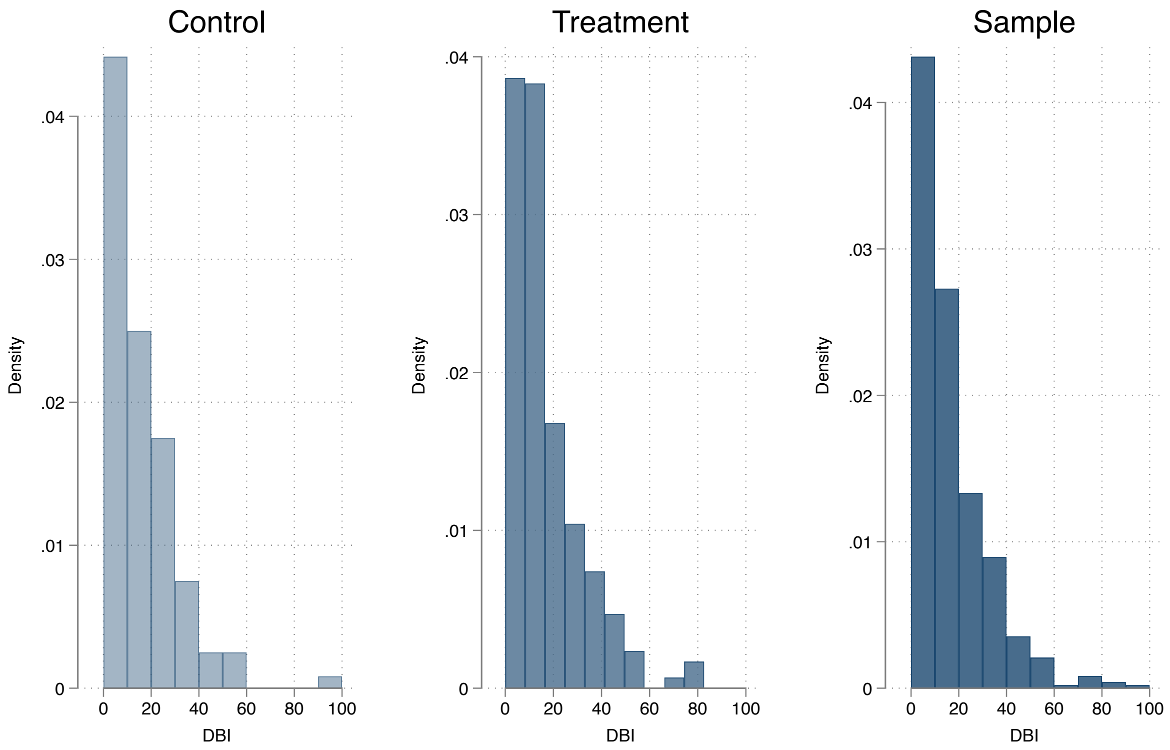

Figure C.3: Distribution of the **Disease Burden Index (DBI)** for Treatment and Control Groups

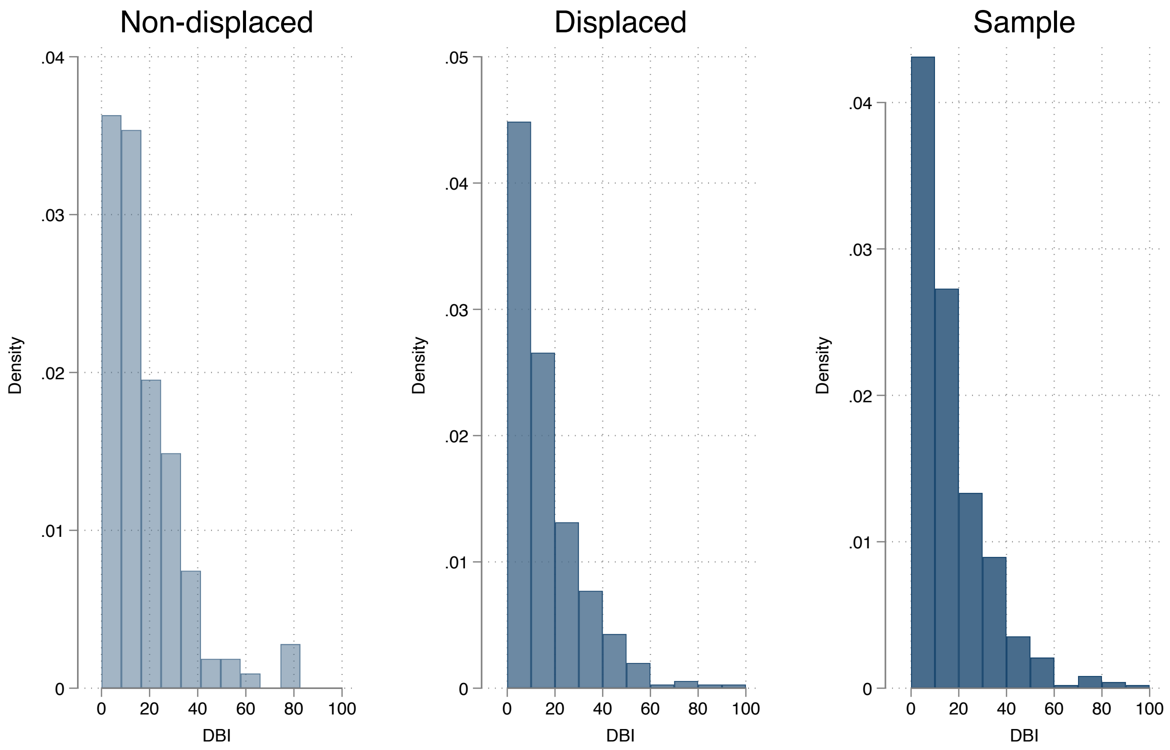

Figure C.4: Distribution of the **Disease Burden Index (DBI)** by Displacement Status

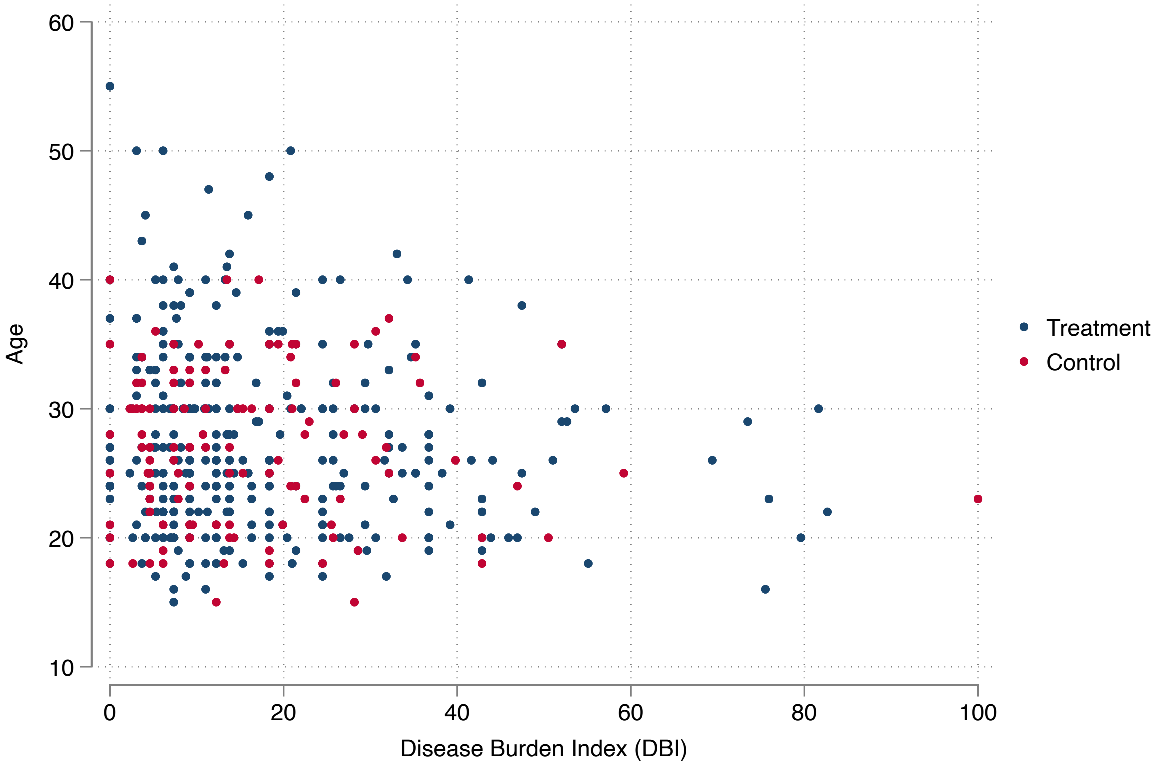

Figure C.5: Age Distribution against the Disease Burden Index (DBI) for Treatment and Control Groups

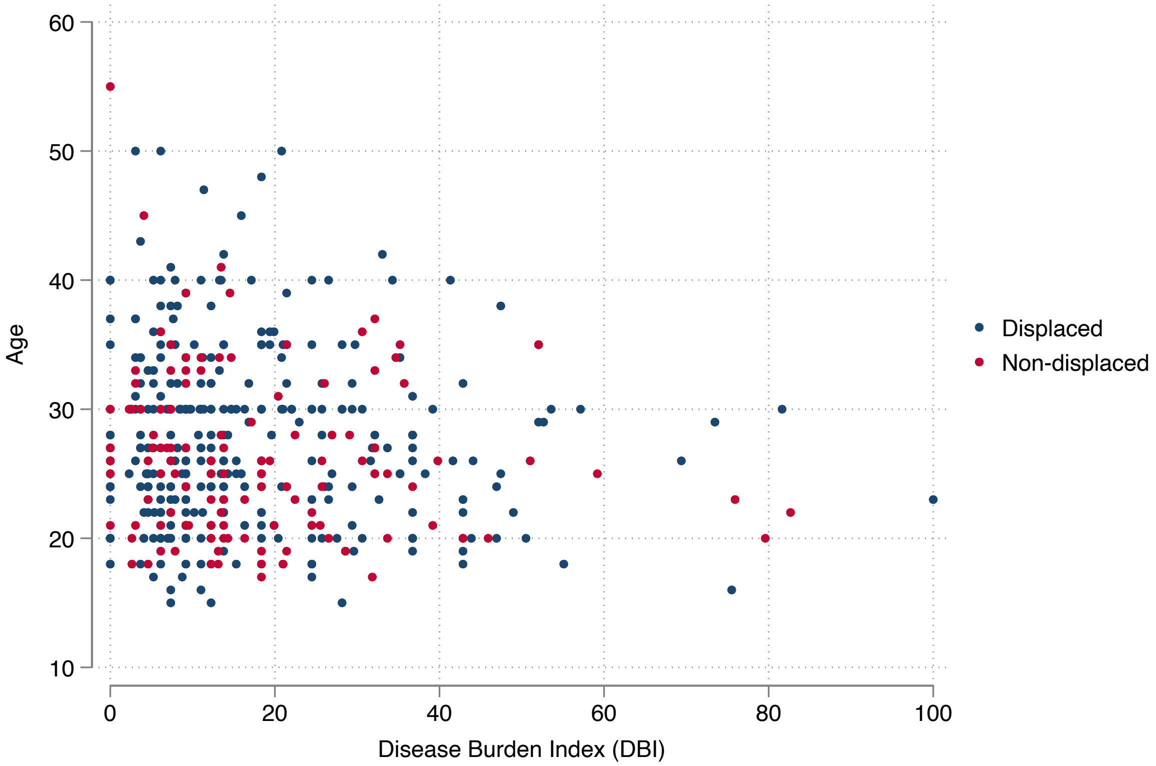

Figure C.6: Age Distribution against the Disease Burden Index (DBI) by Displacement Status

C.2.2 Sensitivity to Alternative DBI Weighting Schemes

To assess whether our findings for Model 1 are driven by the specific disease–severity weights used to construct the Disease Burden Index (DBI), we re-estimated the index under three alternative weighting schemes and re-ran all treatment-effect estimators. In all cases the resulting DBI was re-normalised to the 0–100 scale.

(i) *Equal weights*: all disease categories receive the same weight, so that the index only reflects the number of distinct conditions reported within the household.

(ii) *Compressed severity scale*: very high and high severity conditions are grouped more tightly (e.g. weights in the range 4–5.5 compressed to 4–4.5), reducing the spread between mild and severe conditions.

(iii) *Excluding low-impact conditions*: diseases classified as low impact (e.g. minor skin problems or muscle soreness) are given zero weight, so that the index focuses on moderate and severe

conditions.

Table C.3 reports the Average Treatment Effect (ATE) of displacement on the DBI for each weighting scheme, using the same set of covariates as in Table 3 of the main text. Across all specifications and estimators the ATE remains negative and of similar magnitude, indicating that the lower disease burden among displaced households is not an artefact of the particular DBI weighting scheme.

Table C.3: Sensitivity of ATE Estimates to Alternative DBI Weighting Schemes (Model 1)

**Weighting scheme**	**RA**	**IPW**	**AIPW**	**IPWRA**	**NN**
Baseline severity weights	−4.85	−4.20	−4.19	−4.21	−6.19
Equal weights	−4.40	−3.90	−3.92	−3.95	−5.70
Compressed severity scale	−4.10	−3.65	−3.68	−3.71	−5.30
Excluding low-impact conditions	−5.20	−4.60	−4.55	−4.59	−6.50

Notes: Entries are ATE estimates (in DBI index points) of displacement on the Disease Burden Index (DBI) under different weighting schemes. The first row reproduces the baseline ATEs reported in Table 3 of the main text. The remaining rows use (i) equal weights across disease categories, (ii) a compressed severity scale with less spread between mild and severe conditions, and (iii) zero weight for low-impact conditions. In all cases the DBI is re-scaled to lie between 0 and 100. The ATE remains negative and of similar magnitude across schemes, indicating that the main conclusions are robust to reasonable changes in the DBI weights.

C.3 Wealth Household Index (WHI)

The Wealth Household Index (WHI) is designed to provide a comprehensive measure of a household's economic resources by capturing multiple dimensions of wealth. The index incorporates three primary components. The first component is land ownership, which includes the monetary value of farmland, homestead land, and other rented or mortgaged land, as shown in Table C.4. The second component is the value of household goods, encompassing assets such as furniture, appliances, and means of transportation, as reported in the survey. The final component is animals owned, which accounts for the monetary value of livestock and other animals, such as cows, goats, and poultry, as presented in Table C.4.

These values are then aggregated to compute the Raw Wealth Household Index as follows:

$$\text{Raw WHI} = \frac{(\text{Value of Land} + \text{Value of Goods Owned} + \text{Value of Animals Owned})}{\text{Total Household Members}}. \tag{C.6}$$

To ensure comparability across households, the index is normalized to a standardized range between 0 and 100. The normalization formula is:

$$\text{Normalized WHI} = \frac{\text{Raw WHI Value} - \text{Minimum WHI}}{\text{Maximum WHI} - \text{Minimum WHI}} \times 100., \tag{C.7}$$

where *Minimum WHI* and *Maximum WHI* represent the minimum and maximum observed Wealth Household Index values within the dataset.

This normalization ensures that the Wealth Household Index provides a consistent and comparable measure of economic capacity across all households, making it a reliable indicator for assessing socio-economic disparities in the study population.

Table C.4: Categories and Values for the Wealth Household Index (WHI) in Bangladeshi Taka

**Category**	**Value (in BDT)**
**Land Categories (Values per acre)**	
Own Farmland	50,000
Homestead Land	40,000
Rented Land	30,000
Give Land mortgaged to another	50,000
Take Land mortgaged to another	25,000
**Animal Categories (Values per head)**	
Cow	25,000
Goat	5,000
Sheep	4,000
Buffalo	30,000
Hen	500
Duck	400
Swan	600

For each component, the monetary values are calculated based on the market value of the respective assets. These values are then aggregated to compute the Raw Wealth Household Index as follows:

$$\text{Raw WHI} = \frac{(\text{Value of Land} + \text{Value of Goods Owned} + \text{Value of Animals Owned})}{\text{Total Household Members}}. \tag{C.8}$$

To ensure comparability across households, the index is normalized to a standardized range between 0 and 100. The normalization formula is:

$$\text{Normalized WHI} = \frac{\text{Raw WHI Value} - \text{Minimum WHI}}{\text{Maximum WHI} - \text{Minimum WHI}} \times 100, \tag{C.9}$$

where *Minimum WHI* and *Maximum WHI* represent the minimum and maximum observed Wealth Household Index values within the dataset.

This normalization ensures that the Wealth Household Index provides a consistent and comparable measure of economic capacity across all households, making it a reliable indicator for assessing socio-economic disparities in the study population.

To illustrate the distribution of wealth across the surveyed population, histograms are presented for the WHI, disaggregated by treatment, Figure C.7 and displacement status, Figure C.8 . These histograms allow for a clear comparison of wealth between households in treatment chars (where Friendship NGO operates) and control chars, as well as between displaced and non-displaced households.

To further analyze the relationship between age and wealth, scatter plots of the DBI are presented, stratified by treatment, Figure C.9 and displacement status, Figure C.10. These plots provide insights into how age influences wealth across different groups, capturing potential variations among younger and older household members.

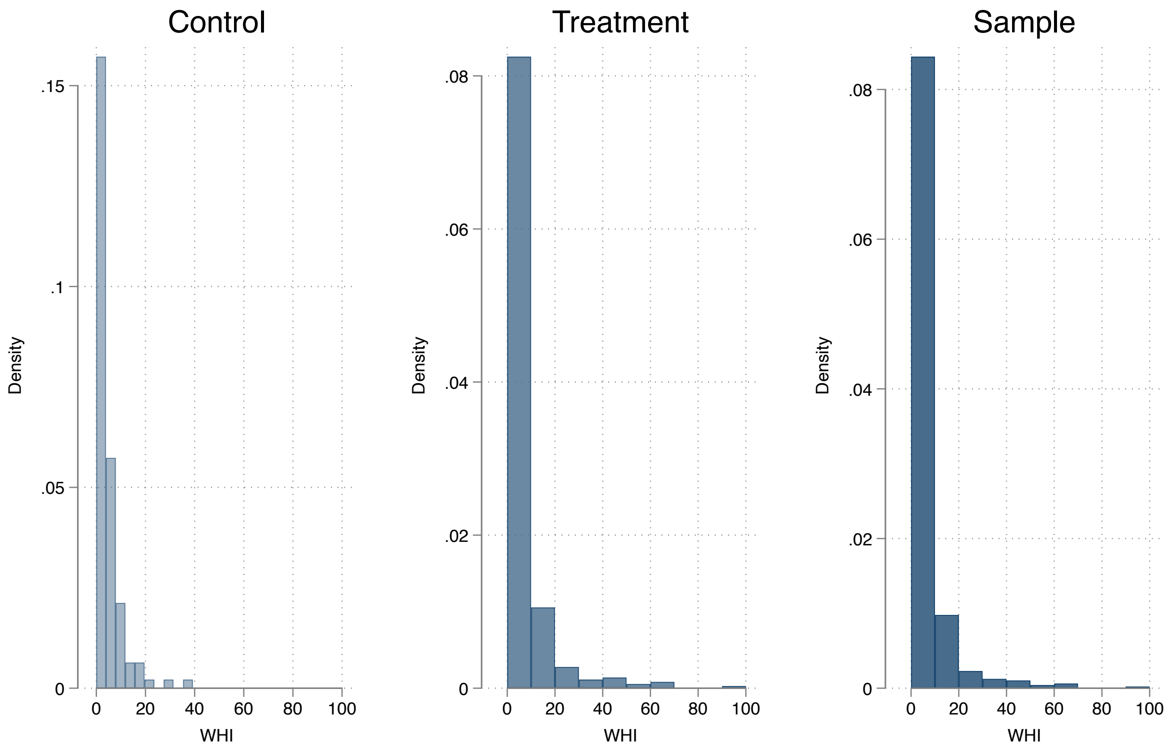

Figure C.7: Distribution of the **Wealth Household Index (WHI)** for Treatment and Control Groups

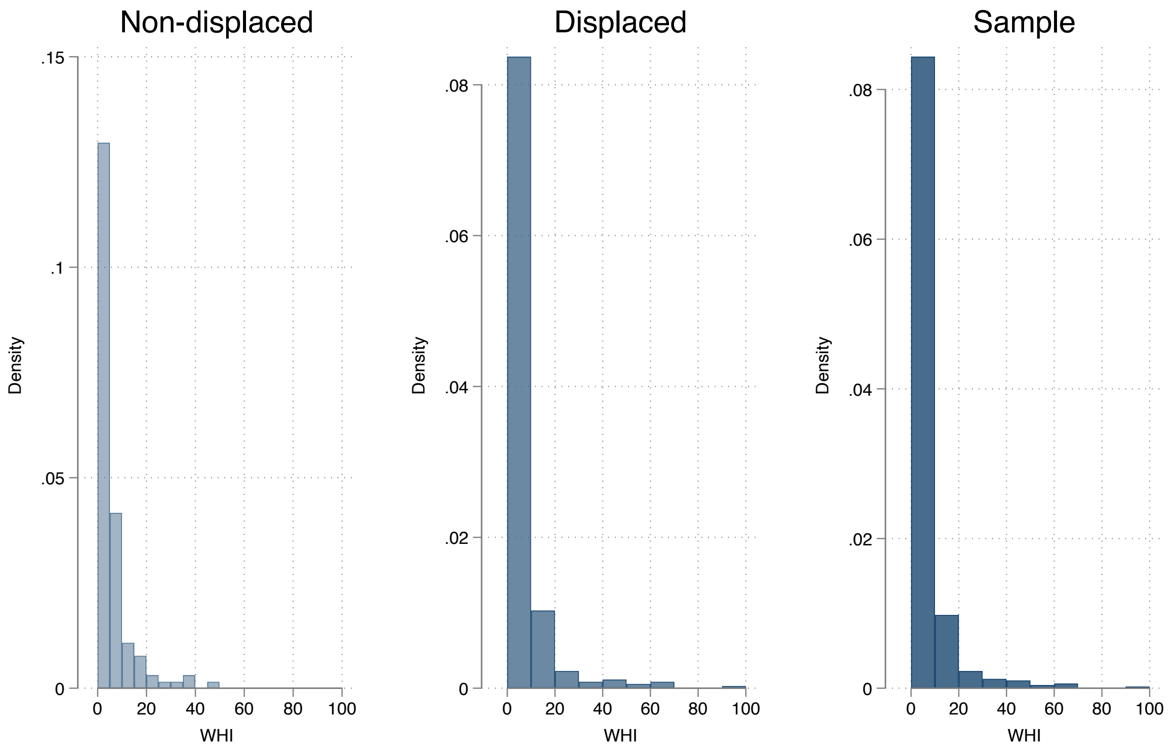

Figure C.8: Distribution of the **Wealth Household Index (WHI)** by Displacement Status

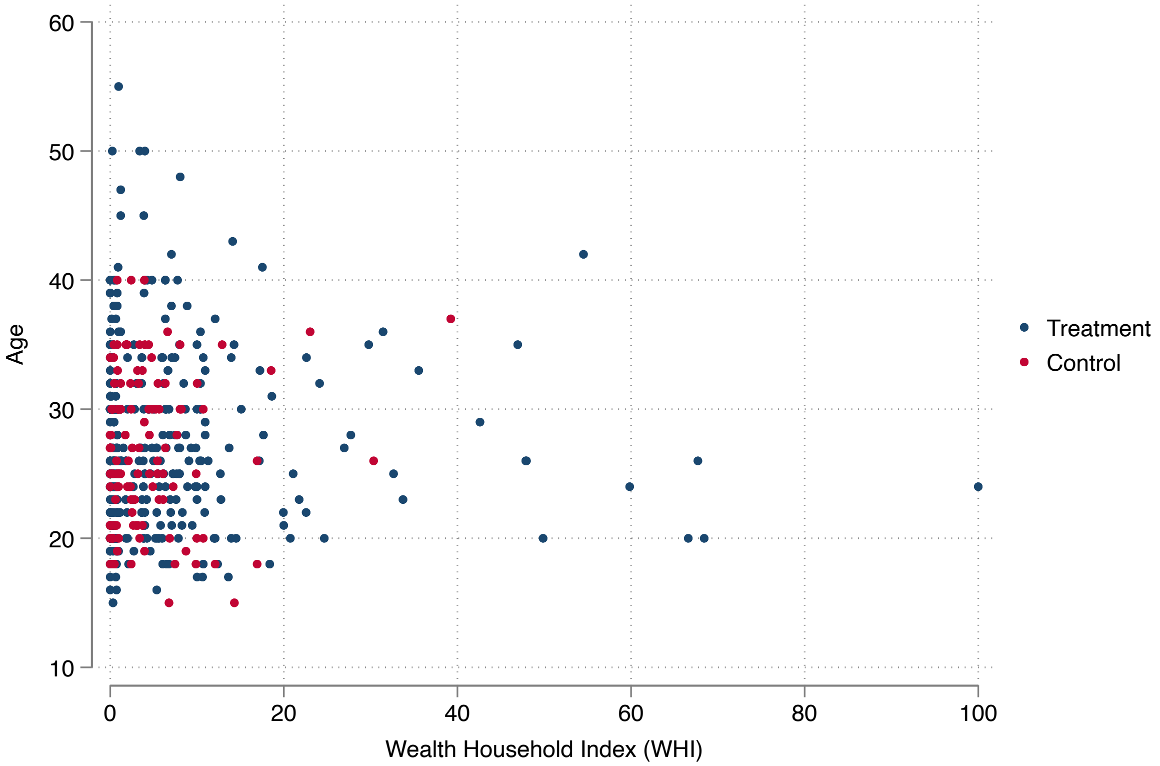

Figure C.9: Age Distribution against the Wealth Household Index (WHI) for Treatment and Control Groups

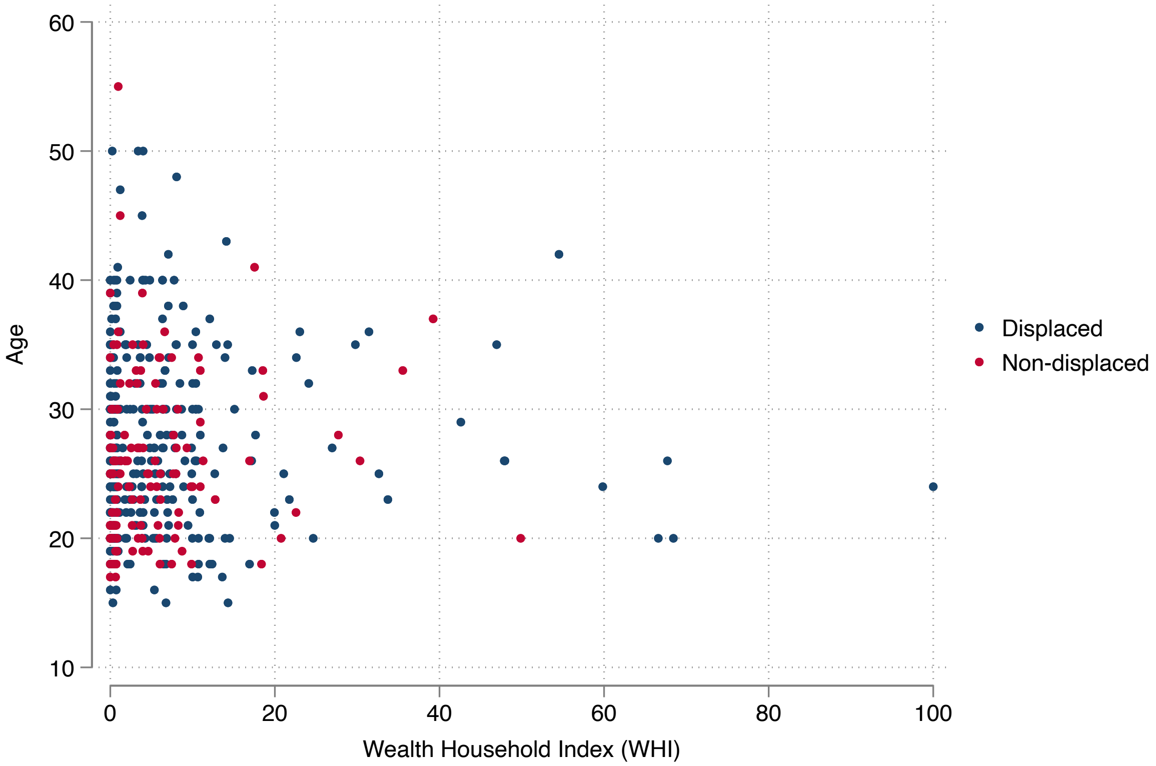

Figure C.10: Age Distribution against the Wealth Household Index (WHI) by Displacement Status

C.4 Hygiene Household Index (HHI)

The Hygiene Household Index (HHI) quantifies household hygiene practices by integrating data on sanitation behaviors, infrastructure, cultural practices, water usage, and accessibility, and toilet facilities. This approach captures the multidimensional nature of hygiene, reflecting both individual actions and the structural and cultural factors that influence them. The index combines binary variables for specific hygiene practices with categorical variables for the main sources of water, the distance to clean water and toilet facilities. The steps for constructing the HHI are outlined below:

C.4.1 Data Components

The dataset encompasses various aspects of hygiene and sanitation, as outlined in Table C.5. It includes binary responses for "Hygiene Practices," assessing behaviors such as handwashing and waste disposal. Additionally, scores were assigned to the "Main Source of Water" based on its ac-

cessibility and reliability, while the "Nearest Source of Water" was evaluated according to proximity and ease of access. "Toilet Facilities" were also scored to reflect sanitation quality.

Table C.5: Hygiene Practices and Related Scores for Water and Sanitation

Category	Details/Score
**Hygiene Practices (Binary Variables)**	
Washing hands with soap before eating	1 = Practice followed, 0 = Not followed
Wearing sandals while entering the latrine	1 = Practice followed, 0 = Not followed
Washing hands with soap or ash after defecation	1 = Practice followed, 0 = Not followed
Washing vegetables and fruits with safe water	1 = Practice followed, 0 = Not followed
Disposing of household waste in designated pits	1 = Practice followed, 0 = Not followed
Using safe water for cooking	1 = Practice followed, 0 = Not followed
Proper disposal of baby feces in holes or toilets	1 = Practice followed, 0 = Not followed
Washing hands with soap after cleaning a baby's stool	1 = Practice followed, 0 = Not followed
Regularly cleaning latrines by pouring water or other means	1 = Practice followed, 0 = Not followed
**Main Source of Water and Scores**	
Hand pump/tube well	5.0
Purchased water	4.0
Open source of water	0.5
Pond Sand Filter (PSF)	2.0
Rainwater Harvesting Plant	2.0
Others	1.0
**Nearest Source of Water and Scores**	
Less than 1 km	2.0
1 km	1.5
1.5 km	1.0
2 km	0.5
Greater than 2 km	0.0
Others	0.5
**Toilet Facilities and Scores**	
Pakka Toilet	5
Only ring-slab toilet	4
Kaccha Toilet	2
Open Place	1
In the bushes	0.2
Others	0.3

C.4.2 Index Calculation

The HHI is calculated as follows: The hygiene score is computed by summing the binary variables and the scores for the main source and nearest source of water:

$$\text{Hygiene Score} = \frac{(\sum_{i=1}^{9} \text{Binary Variable}_i + \text{Water Source Score} + \text{Distance Score} + \text{Toilet Facility})}{\text{Total Household Members}}. \tag{C.10}$$

To ensure the index ranges between 0 and 100, the hygiene score is normalized by dividing by the maximum possible score and multiplying by 100:

$$\text{HHI} = \frac{\text{Hygiene Score}}{\text{Maximum Possible Score}} \times 100. \tag{C.11}$$

The maximum possible score is calculated as the sum of the maximum scores for the binary variables (9), the water source score (5), the distance score (2), and the toilet facility (5), resulting in a total of 21. By scaling the normalized value to a range of 0 to 100, the HHI becomes more intuitive and comparable across households. A higher HHI value indicates better hygiene practices, safer water sources, and greater accessibility. This index provides a comprehensive measure for evaluating household hygiene and identifying areas in need of targeted interventions.

To explore hygiene practices across different household groups, histograms of the HHI are presented, disaggregated by treatment, Figure C.11 and displacement status, Figure C.12. These visualizations allow for a clear comparison of hygiene conditions between households in treatment chars (beneficiaries of Friendship NGO interventions) and control chars, as well as between displaced and non-displaced households.

To analyze how age influences hygiene-related practices, scatter plots of the HHI are presented, stratified by treatment, Figure C.13 and displacement status, Figure C.14. These plots visually depict the relationship between the HHI and the age of household members, offering insights into whether younger or older household members face greater hygiene challenges.

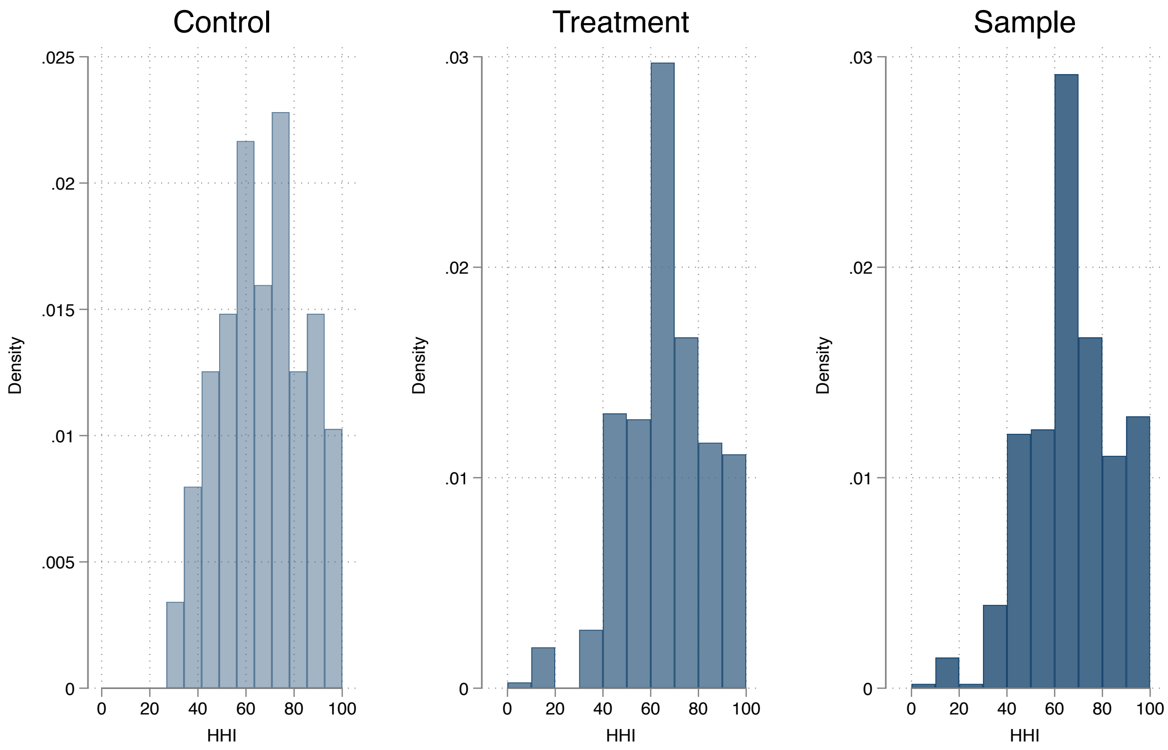

Figure C.11: Distribution of the **Hygiene Household Index (HHI)** for Treatment and Control Groups

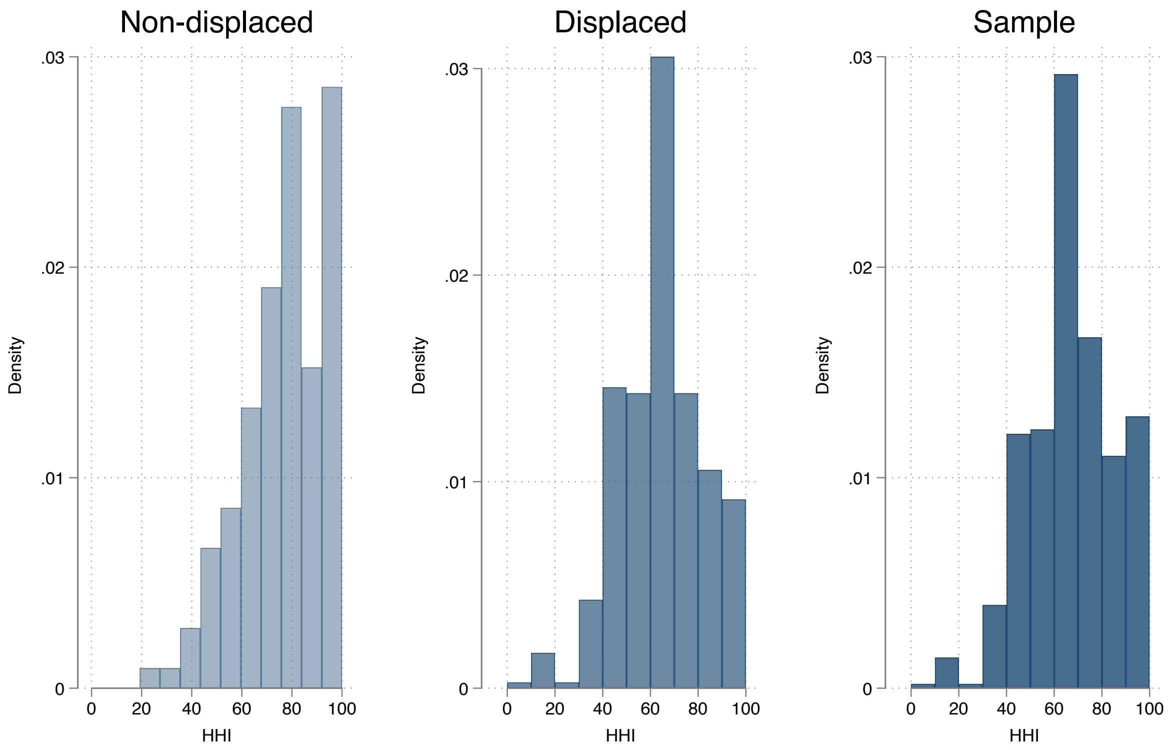

Figure C.12: Distribution of the **Hygiene Household Index (HHI)** by Displacement Status

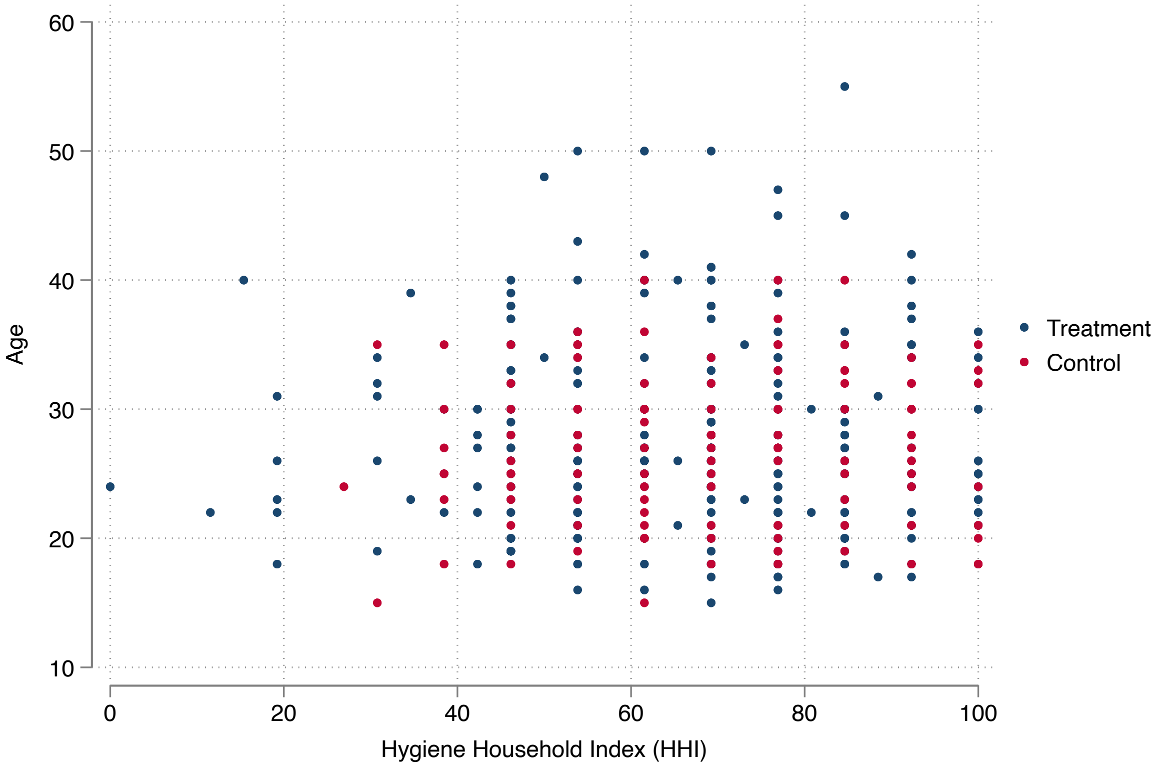

Figure C.13: Age Distribution against the Hygiene Household Index (HHI) for Treatment and Control Groups

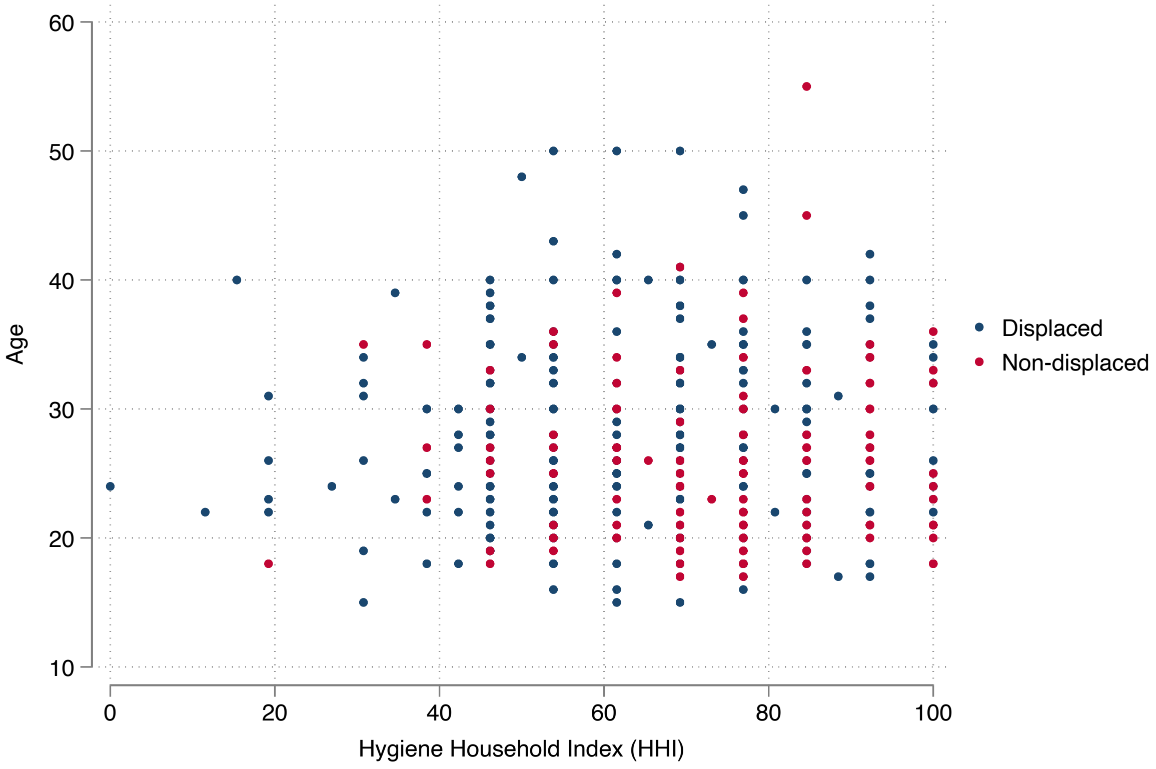

Figure C.14: Age Distribution against the Hygiene Household Index (HHI) by Displacement Status

C.5 Educational Attainment Index (EAI)

The Educational Attainment Index (EAI) is designed to measure the overall educational achievement within a household, incorporating both the highest level of education attained by each member and their age. This scaling ensures that the index reflects the educational outcomes relative to the population's demographics.

Each household member is assigned a weight based on their highest level of education, as shown in Table C.6. These weights are further scaled by the age of each individual to reflect the age-adjusted educational capacity of the household. This approach captures variations in educational attainment across different age groups while providing a meaningful comparison across households.

Table C.6: Weights Assigned to Educational Levels in the EAI

Educational Level	Weight
Illiterate	0
Can sign	1
Primary class (1–4)	2
PEC (Passed Class 5)	3
JSC (Passed Class 8)	4
SSC	5
HSC	6
Degree	7
Honors	8
Masters	9
Others (non-traditional education)	5
Child (below schooling age)	0

The raw Educational Attainment Index is calculated as:

$$\text{Raw EAI} = \frac{\sum_{i=1}^{N} (\text{Weight}_i \times \text{Age}_i)}{\text{Total Household Members}}, \tag{C.12}$$

where N is the total number of household members, Weight_i is the educational weight for the i-th individual, and Age_i is their age. This formulation scales the educational weight by the individual's age to provide an age-adjusted score.

To standardize the EAI across households, the raw index is normalized and scaled to fall between 0 and 100:

$$\text{EAI} = \frac{\text{Raw EAI} - \text{Minimum EAI}}{\text{Maximum EAI} - \text{Minimum EAI}} \times 100. \tag{C.13}$$

Normalization ensures that the EAI is comparable across households of varying sizes and demographics, with final values ranging from 0 to 100–higher scores indicate greater age-adjusted educational achievement. This adjustment reflects the differing contributions of individuals based on their educational attainment and life stage, capturing both realized and potential impacts.

Histograms of the EAI are presented, disaggregated by treatment, Figure C.15 and displacement status, Figure C.16. These histograms provide a comparative view of educational attainment between households in treatment chars (where Friendship NGO operates) and control chars, as well as between displaced and non-displaced households.

To examine the relationship between age and educational attainment, scatter plots of the EAI are presented, stratified by treatment, Figure C.17 and displacement status, Figure C.18. These plots illustrate how education levels vary with age, highlighting generational differences in access to education.

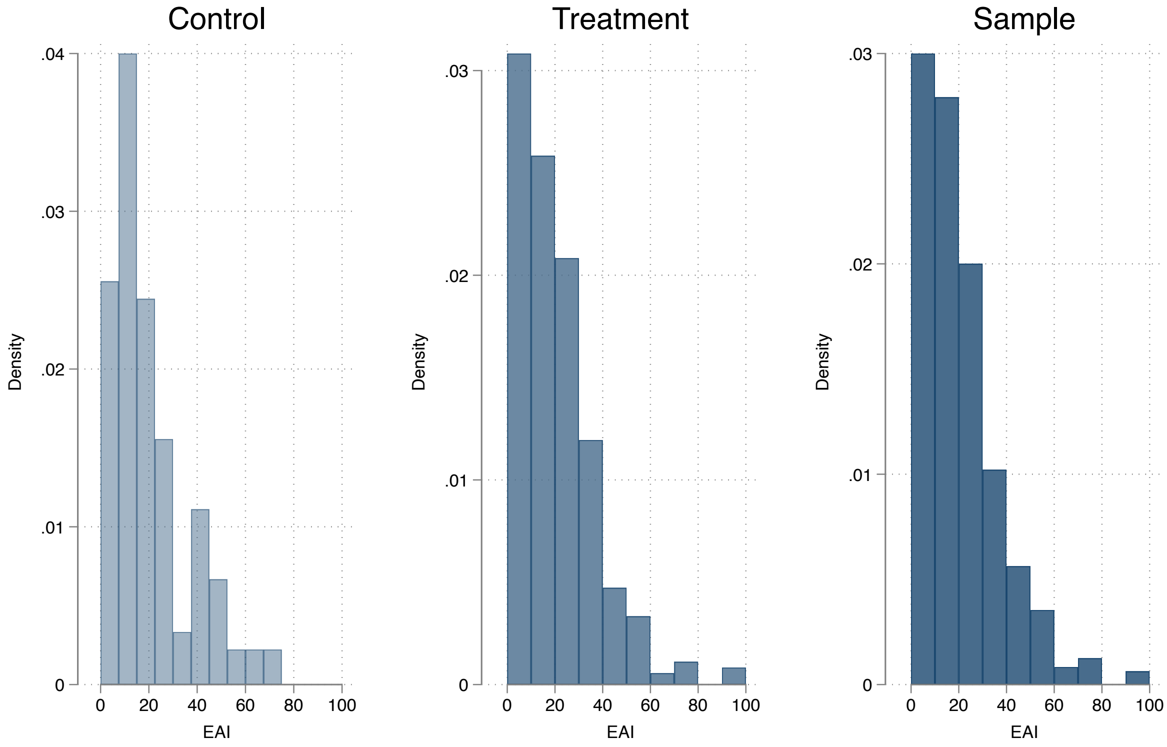

Figure C.15: Distribution of the **Education Attainment Index (EAI)** for Treatment and Control Groups

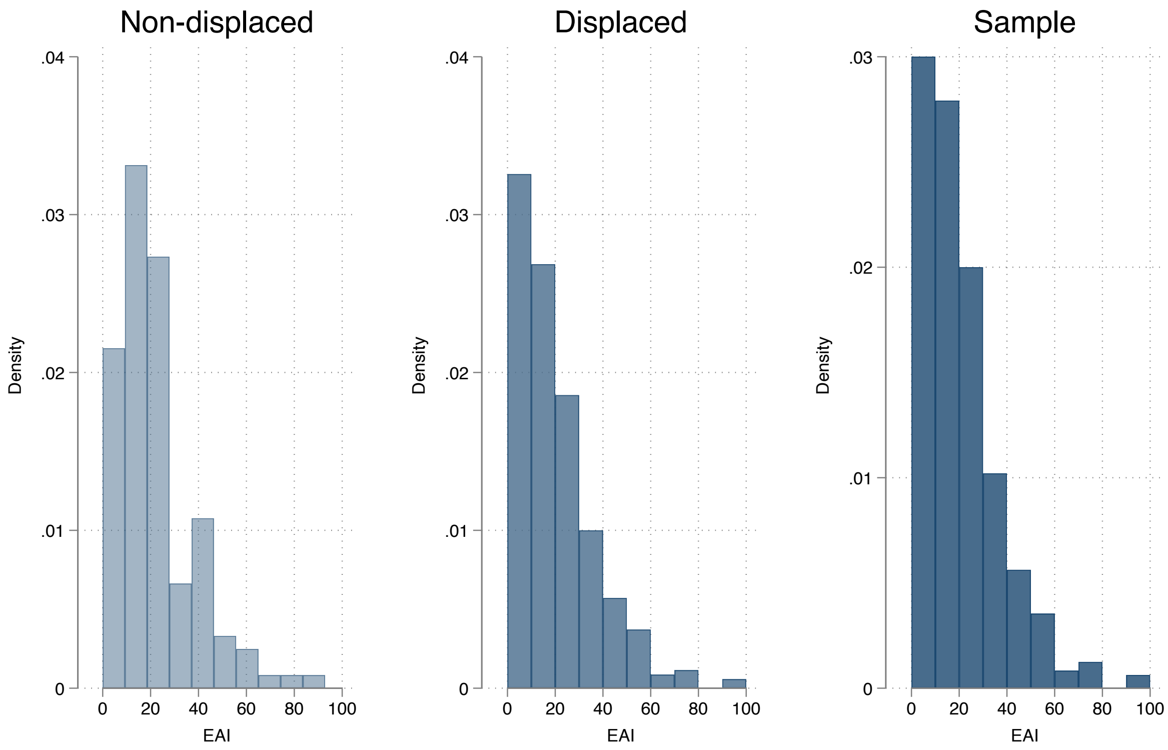

Figure C.16: Distribution of the **Education Attainment Index (EAI)** by Displacement Status

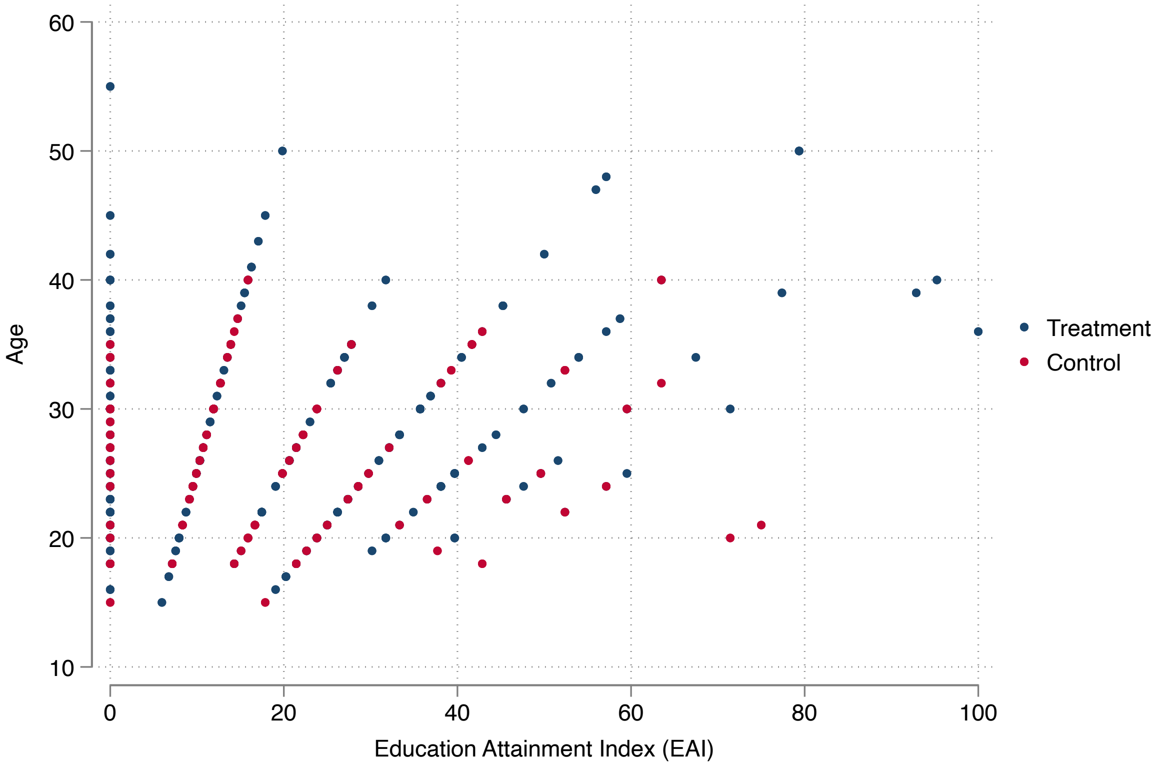

Figure C.17: Age Distribution against the Education Attainment Index (EAI) for Treatment and Control Groups

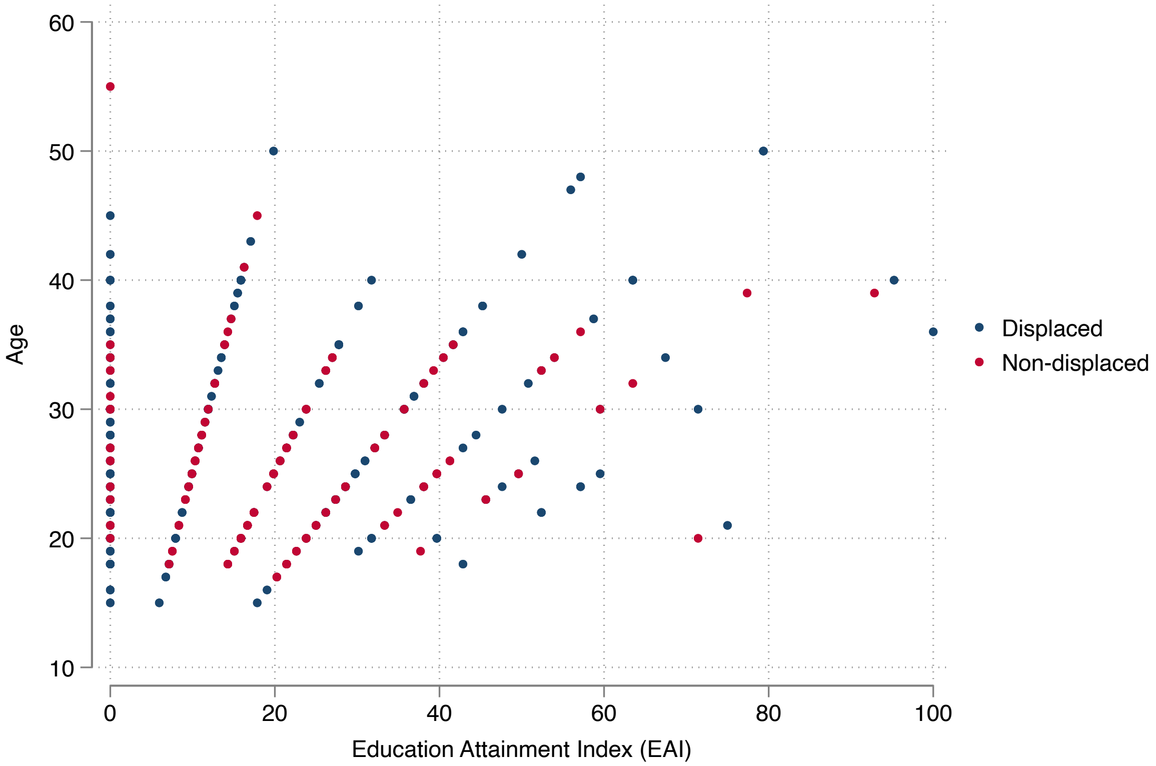

Figure C.18: Age Distribution against the Education Attainment Index (EAI) by Displacement Status

C.6 Summary statistics for the Indexes

The survey results are summarized through the construction of five key indices – Disease Burden Index (DBI), Wealth Household Index (WHI), Migration Household Index (MHI), Hygiene Household Index (HHI), and Educational Attainment Index (EAI) – to evaluate health and socio-economic conditions. Table C.7 and Table C.8 presents the descriptive statistics of these indices, providing a comparative overview of the treatment and control chars, as well as displaced and non-displaced households, to highlight key patterns and disparities.

Table C.7: Descriptive Statistics for Indexes by Treatment and Control Groups

Index	Group	Mean	p10	p25	p50	p75	p90	SD	Min	Max	N
	Control	16.07	3.37	5.69	12.24	21.94	32.14	14.57	0.00	100.00	120
**DBI**	Treatment	17.03	3.67	7.35	12.24	24.49	36.73	15.05	0.00	82.65	360
	Total	16.79	3.67	6.94	12.24	23.72	36.73	14.92	0.00	100.00	480
	Control	4.32	0.00	0.58	2.47	5.63	9.99	5.90	0.00	39.23	120
**WHI**	Treatment	6.36	0.00	0.38	1.99	7.53	14.18	11.65	0.00	100.00	360
	Total	5.85	0.00	0.40	2.42	7.01	13.25	10.54	0.00	100.00	480
	Control	16.29	3.42	7.69	11.97	20.52	35.90	12.24	0.00	55.56	65
**MHI**	Treatment	21.47	4.27	9.83	17.95	29.49	42.74	15.85	0.00	100.00	284
	Total	20.51	4.27	9.40	17.10	28.21	42.74	15.36	0.00	100.00	349
	Control	68.94	46.15	53.85	69.23	84.62	92.31	18.48	26.92	100.00	120
**HHI**	Treatment	67.22	46.15	53.85	69.23	76.92	92.31	17.94	0.00	100.00	360
	Total	67.65	46.15	53.85	69.23	76.92	92.31	18.07	0.00	100.00	480
	Control	20.03	0.00	9.52	15.48	27.58	44.25	17.18	0.00	75.00	120
**EAI**	Treatment	19.79	0.00	7.94	15.87	28.57	41.07	17.71	0.00	100.00	360
	Total	19.85	0.00	8.73	15.87	28.57	41.67	17.56	0.00	100.00	480

Table C.8: Summary Statistics for Displaced vs. Non-Displaced Households

Index	Group	Mean	p10	p25	p50	p75	p90	SD	Min	Max	N
**DBI**	Non-Displaced	17.73	3.06	7.35	13.47	24.49	34.95	15.38	0.00	82.65	130
	Displaced	16.44	3.67	6.12	11.37	22.04	36.73	14.75	0.00	100.00	350
	Total	16.79	3.67	6.94	12.24	23.72	36.73	14.92	0.00	100.00	480
**WHI**	Non-Displaced	5.49	0.04	0.64	2.93	7.46	12.02	8.02	0.00	49.87	130
	Displaced	5.98	0.00	0.32	1.98	6.97	13.66	11.34	0.00	100.00	350
	Total	5.85	0.00	0.40	2.42	7.01	13.25	10.54	0.00	100.00	480
**MHI**	Non-Displaced	–	–	–	–	–	–	–	–	–	0
	Displaced	20.51	4.27	9.40	17.10	28.21	42.74	15.36	0.00	100.00	349
	Total	20.51	4.27	9.40	17.10	28.21	42.74	15.36	0.00	100.00	349
**HHI**	Non-Displaced	74.41	53.85	61.54	76.92	84.62	92.31	16.81	19.23	100.00	130
	Displaced	65.14	46.15	53.85	69.23	76.92	84.62	17.91	0.00	100.00	350
	Total	67.65	46.15	53.85	69.23	76.92	92.31	18.07	0.00	100.00	480
**EAI**	Non-Displaced	21.34	0.00	10.32	17.66	26.98	41.27	16.89	0.00	92.86	130
	Displaced	19.30	0.00	7.14	15.28	28.57	42.86	17.80	0.00	100.00	350
	Total	19.85	0.00	8.73	15.87	28.57	41.67	17.56	0.00	100.00	480

D Multicollinearity Diagnostics

To ensure that our results are not driven by multicollinearity among the covariates, we computed Variance Inflation Factors (VIFs) for the full set of regressors used in Models 1 and 2. Multicollinearity inflates the variance of coefficient estimates, potentially leading to unstable inference; a common rule of thumb is that VIF values above 10 indicate serious multicollinearity problems (2–4).

Table D.1 reports VIFs for the main indices and selected controls. All VIF values are well below the conventional threshold of 10, and the mean VIF is below 2, indicating that multicollinearity is not a concern for our treatment-effect estimates.

Table D.1: Variance Inflation Factor (VIF) Results

Variable	Category / Level	VIF	1/VIF
**HHI**	Hygiene Household Index	1.19	0.8427
**MHI**	Migration Household Index	1.36	0.7376
**DBI**	Disease Burden Index	1.22	0.8221
**WHI**	Wealth Household Index	1.24	0.8082
**EAI**	Educational Attainment Index	1.26	0.7937
**Age**	Respondent age	1.16	0.8602
**Non-agriculture income (quintile dummies)**			
Quantile 2		1.65	0.6067
Quantile 3		1.62	0.6188
Quantile 4		1.35	0.7418
Quantile 5		1.42	0.7024
**Agriculture income (quintile dummies)**			
Quantile 2		1.68	0.5945
Quantile 3		2.01	0.4968
Quantile 4		1.61	0.6202
Quantile 5		1.50	0.6654
**Char fixed effects**			
Batikamari		2.47	0.4054
Char Jatrapur		3.59	0.2787
Chor Garuhara		2.51	0.3986
Datiar Char		1.88	0.5333
Kawa Bada		3.23	0.3100
Khamar Bashpata		2.71	0.3694
Khamar Holokhana		2.74	0.3653
Kheyar Alga		2.44	0.4093
Korai Barishal		2.13	0.4695
Moheshbandi		2.30	0.4339
South Sannasir Char		2.39	0.4182
**Mean VIF**		**1.94**	

Notes: VIFs are based on the pooled covariate set used in Models 1 and 2. For groups of dummies (income quintiles and char indicators), the table reports separate VIFs for each category. All values lie well below the commonly used threshold of 10, and the mean VIF is below 2, indicating limited multicollinearity.

E Covariate Balance and Propensity-Score Overlap

In addition to the VIF diagnostics, we assess covariate balance between treated and control groups using standardised mean differences (SMDs) and the overlap of propensity-score distributions for both models.

For a generic covariate X, the SMD is defined as

$$\text{SMD} \;=\; \frac{\bar{X}_T - \bar{X}_C}{\sqrt{\frac{1}{2}\left(s_T^2 + s_C^2\right)}},$$

where $\bar{X}_T$ and $\bar{X}_C$ are the treated and control means and s_T^2, s_C^2 the corresponding variances. Values with $|\text{SMD}| < 0.1$ are typically interpreted as indicating good balance.

We report SMDs for the main covariates before reweighting, after applying the IPW/IPWRA weights, and for the matched samples used in the nearest-neighbour (NN) estimator.

Table E.1: Standardised Mean Differences (SMDs) Before and After Weighting / Matching

Model	Covariate	Unweighted SMD	Weighted SMD	Matched SMD
1 (DBI / displacement)	WHI	0.28	0.035	0.009
	HHI	0.23	0.028	0.007
	EAI	0.18	0.021	0.006
	Age	0.11	0.026	0.001
	Agric. income Q4	0.28	0.022	0.008
	Non-agric. income Q4	0.30	0.026	0.009
2 (MHI / treatment char)	WHI	0.31	0.038	0.012
	HHI	0.27	0.030	0.010
	EAI	0.22	0.025	0.008
	Age	0.15	0.032	0.006
	Agric. income Q4	0.33	0.041	0.015
	Non-agric. income Q4	0.29	0.036	0.013

Notes: SMDs are defined as the difference in covariate means between treated and control groups, divided by the pooled standard deviation. Values below 0.1 in absolute value are typically taken as indicating good balance. In our application, several covariates display $|\text{SMD}| > 0.2$ in the unweighted sample, while all reported covariates satisfy $|\text{SMD}| < 0.1$ after weighting or matching in both models.

For each model, we also estimate a propensity-score model using logistic regression. In Model 1 the dependent variable is displacement status (displaced vs. non-displaced), and in Model 2 it indicates residence in a treatment char (Friendship vs. control). The covariate vector X_i includes the five household indices (DBI or MHI, WHI, HHI, EAI), respondent age, agricultural and non-agricultural income quintiles, and char fixed effects.

Figures E.1 and E.2 display the estimated propensity-score distributions by treatment status for Model 1 and Model 2, respectively. In both cases there is substantial common support between treated and control groups, with relatively few observations in the extreme tails, supporting the positivity (overlap) assumption required for weighting and matching estimators.

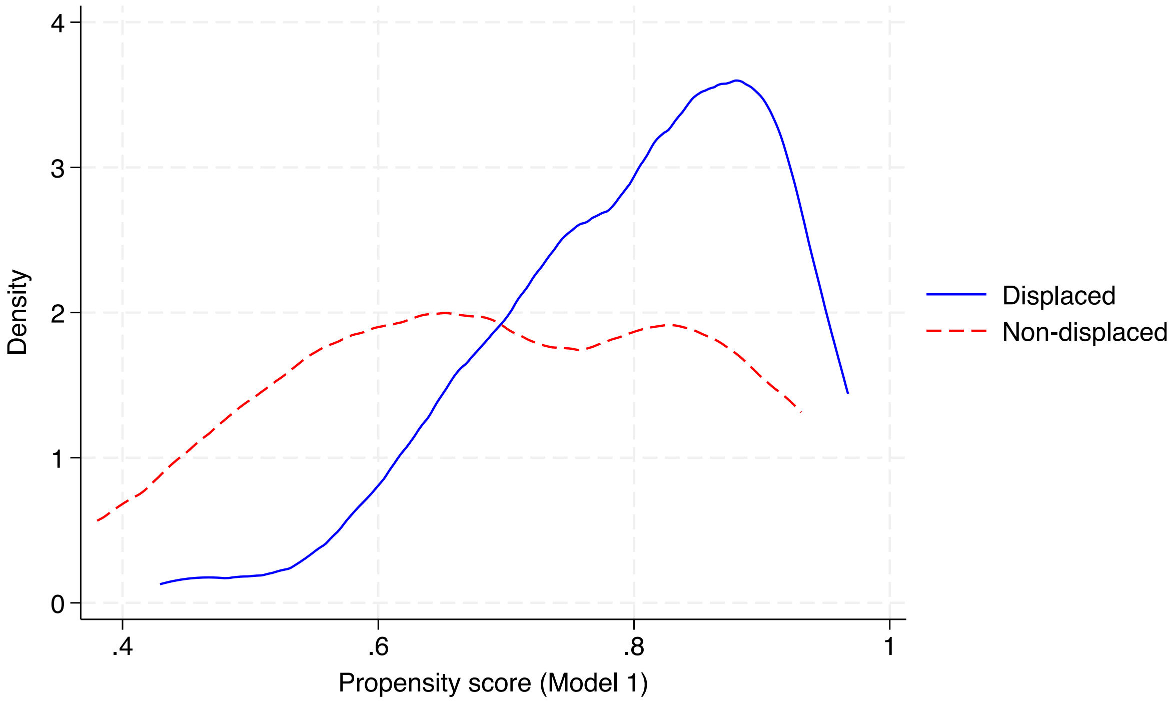

Figure E.1: Model 1 – Estimated Propensity-Score Distributions for Displaced and Non-Displaced Households

Notes: Kernel density estimates of the estimated propensity score for displaced (solid line) and non-displaced (dashed line) households in Model 1. There is substantial common support over the range of propensity scores (roughly 0.45–0.95), with only a small number of observations in the extreme tails. We therefore do not trim any observations. Excluding the few units with scores below 0.50 or above 0.95 leaves the ATE estimates for Model 1 essentially unchanged.

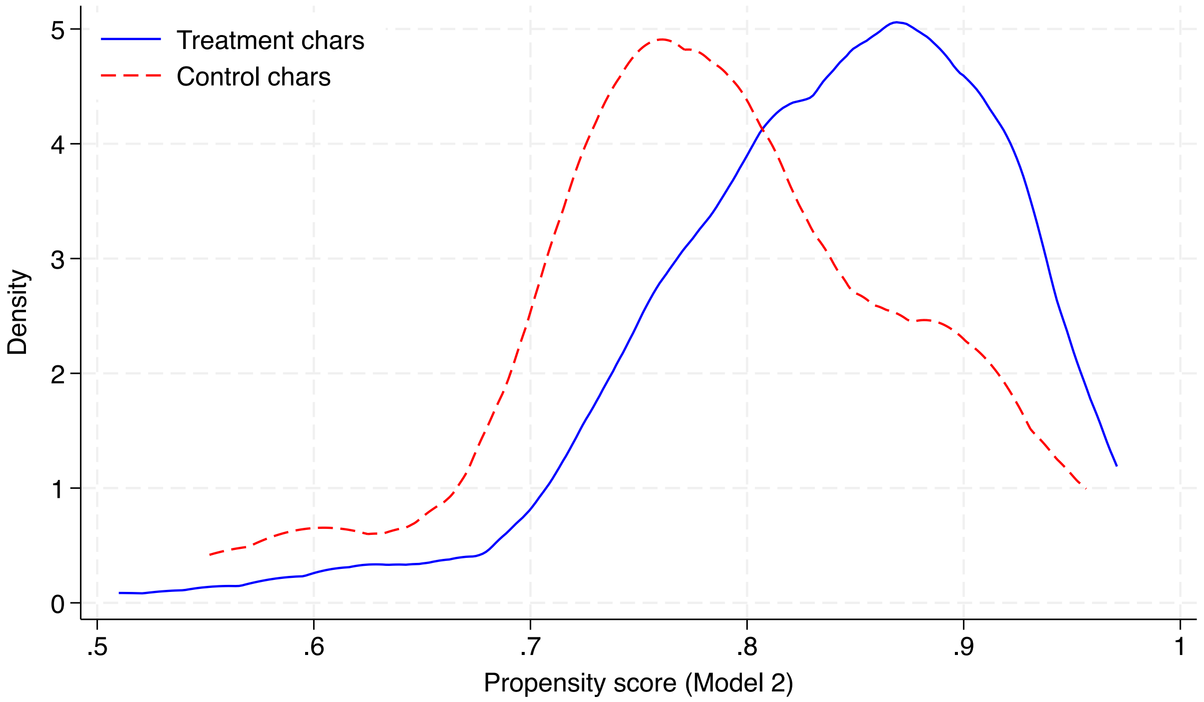

Figure E.2: Model 2 – Estimated Propensity-Score Distributions for Treatment and Control Chars

Notes: Kernel density estimates of the estimated propensity score for households in treatment (solid line) and control (dashed line) chars in Model 2. There is substantial common support over the range of propensity scores (roughly 0.55–0.95), with only a few observations in the extreme tails. We therefore do not trim any observations; excluding the few units with scores below 0.60 or above 0.95 leaves the ATE estimates for Model 2 essentially unchanged.

Inspecting minimum and maximum propensity scores by treatment status confirms that only a very small number of units lie in the tails where overlap is weak. Dropping these extreme observations produces ATE estimates that are virtually identical to those reported in the main text. For transparency, we therefore present results without trimming but note that our conclusions are robust to excluding these few outliers.

F Theoretical Framework Methodology

Given the absence of randomisation, we rely on quasi-experimental methods to estimate causal effects under the standard potential-outcomes framework. Let $D_i \in \{0, 1\}$ denote treatment status (displacement vs. non-displacement in Model 1; residence in a treatment vs. control char in Model 2), and let $Y_i(1)$ and $Y_i(0)$ be the corresponding potential outcomes. The object of interest is the Average

Treatment Effect (ATE),

$$\text{ATE} = E\big[Y_i(1) - Y_i(0)\big],$$

which we identify under the usual assumptions of conditional ignorability and overlap:

$$\{Y_i(1), Y_i(0)\} \perp D_i \mid X_i, \qquad 0 < P(D_i = 1 \mid X_i) < 1$$

for all X_i in the support of the covariates.

We estimate the ATE using five complementary estimators implemented via `teffects` in Stata: Regression Adjustment (RA), Inverse Probability Weighting (IPW), Augmented IPW (AIPW), Inverse Probability-Weighted Regression Adjustment (IPWRA) and nearest-neighbour matching (NN). The covariate vector X_i includes the household indices (DBI or MHI, WHI, HHI, EAI), respondent age, agricultural and non-agricultural income quintiles, and char fixed effects.

Regression Adjustment (RA)

RA specifies an outcome regression model

$$E[Y_i \mid D_i, X_i] = m(D_i, X_i; \beta),$$

typically linear in parameters. Separate predictions $\widehat{Y}_i(1) = m(1, X_i; \widehat{\beta})$ and $\widehat{Y}_i(0) = m(0, X_i; \widehat{\beta})$ are obtained for all units, and the ATE is estimated as the sample average of the difference:

$$\widehat{\text{ATE}}_{\text{RA}} = \frac{1}{N}\sum_{i=1}^{N}\big(\widehat{Y}_i(1) - \widehat{Y}_i(0)\big).$$

Inverse Probability Weighting (IPW)

IPW first models the propensity score $p(X_i) = P(D_i = 1 \mid X_i)$, here using a logit specification

$$p(X_i; \gamma) = \Pr(D_i = 1 \mid X_i) = \frac{\exp(X_i'\gamma)}{1 + \exp(X_i'\gamma)}.$$

The ATE is then estimated by weighting each observation by the inverse of its probability of receiving the treatment it actually received:

$$\widehat{\text{ATE}}_{\text{IPW}} = \frac{1}{N}\sum_{i=1}^{N}\left[\frac{D_i Y_i}{\widehat{p}(X_i)} - \frac{(1-D_i)Y_i}{1-\widehat{p}(X_i)}\right].$$

Augmented IPW (AIPW)

AIPW combines IPW with an outcome regression, yielding a doubly-robust estimator that is consistent if either the propensity-score model or the outcome model is correctly specified:

$$\widehat{\text{ATE}}_{\text{AIPW}} = \frac{1}{N}\sum_{i=1}^{N}\left\{\left[\frac{D_i}{\widehat{p}(X_i)} - \frac{1-D_i}{1-\widehat{p}(X_i)}\right]\big(Y_i - \widehat{m}(D_i, X_i)\big) + \widehat{m}(1, X_i) - \widehat{m}(0, X_i)\right\},$$

where $\widehat{m}(d, X_i)$ denotes fitted values from the outcome regression model.

IPW Regression Adjustment (IPWRA)

IPWRA re-weights the sample by the inverse propensity score and then estimates separate outcome regressions in the treated and control groups. It combines the advantages of RA and IPW and is also doubly robust. In practice we use the `teffects ipwra` estimator in Stata, which jointly estimates the propensity-score and outcome models and reports ATEs and robust standard errors.

Nearest-Neighbour Matching (NN)

Finally, we implement nearest-neighbour matching estimators based on the propensity score. For each treated unit, we find one or more control units with the closest propensity score and compute the average outcome difference across matches. Denoting $\mathcal{C}(i)$ the set of matched controls for treated unit i, the ATT (and by symmetry the ATE in our setting) can be written as

$$\widehat{\text{ATT}}_{\text{NN}} = \frac{1}{N_T}\sum_{i:D_i=1}\left(Y_i - \frac{1}{|\mathcal{C}(i)|}\sum_{j\in\mathcal{C}(i)} Y_j\right),$$

where N_T is the number of treated units. We use propensity-score matching with replacement and robust standard errors to account for the matching procedure.

Taken together, these five estimators provide a rich set of robustness checks: RA relies mainly on the functional form of the outcome model, IPW on the propensity-score model, AIPW and IPWRA are doubly robust, and NN matching requires fewer parametric assumptions but relies on good overlap in the propensity score, documented in Appendix E.

References

[1] Dureja A, Negi DS. Smoothing consumption in times of illness: Household recourse mechanisms. Health Economics. 2024;n/a(n/a). Available from: https://onlinelibrary.wiley.com/doi/abs/10.1002/hec.4830.

[2] Belsley DA. Conditioning diagnostics. Encyclopedia of Statistical Sciences. 2004;2.

[3] Chatterjee S, Hadi AS. Regression analysis by example. John Wiley & Sons; 2015.

[4] Kutner MH, Nachtsheim CJ, Neter J, Li W. Applied linear statistical models. McGraw-hill; 2005.
